# Group VR experiences can produce ego attenuation and connectedness comparable to psychedelics

**DOI:** 10.1038/s41598-022-12637-z

**Published:** 2022-05-30

**Authors:** David R. Glowacki, Rhoslyn Roebuck Williams, Mark D. Wonnacott, Olivia M. Maynard, Rachel Freire, James E. Pike, Mike Chatziapostolou

**Affiliations:** 1 ArtSci International Foundation, Bristol, UK; 2 Citius Intelligent Technologies Research Centre, Santiago de Compostela, Spain; 3 Bristol, UK; 4 School of Psychological Science, University of Bristol, Bristol, UK; 5 Rachel Freire Studio, London, UK

**Keywords:** Sensory processing, Perception

## Abstract

With a growing body of research highlighting the therapeutic potential of experiential phenomenology which diminishes egoic identity and increases one’s sense of connectedness, there is significant interest in how to elicit such ‘self-transcendent experiences’ (STEs) in laboratory contexts. Psychedelic drugs (YDs) have proven particularly effective in this respect, producing subjective phenomenology which reliably elicits intense STEs. With virtual reality (VR) emerging as a powerful tool for constructing new perceptual environments, we describe a VR framework called ‘Isness-distributed’ (Isness-D) which harnesses the unique affordances of distributed multi-person VR to blur conventional self-other boundaries. Within Isness-D, groups of participants co-habit a shared virtual space, collectively experiencing their bodies as luminous energetic essences with diffuse spatial boundaries. It enables moments of ‘energetic coalescence’, a new class of embodied intersubjective experience where bodies can fluidly merge, enabling participants to include multiple others within their self-representation. To evaluate Isness-D, we adopted a citizen science approach, coordinating an international network of Isness-D 'nodes'. We analyzed the results (N = 58) using 4 different self-report scales previously applied to analyze subjective YD phenomenology (the inclusion of community in self scale, ego-dissolution inventory, communitas scale, and the MEQ30 mystical experience questionnaire). Despite the complexities associated with a distributed experiment like this, the Isness-D scores on all 4 scales were statistically indistinguishable from recently published YD studies, demonstrating that distributed VR can be used to design intersubjective STEs where people dissolve their sense of self in the connection to others.

## Introduction

Describing the sensations that arose immediately following a left-hemisphere stroke, brain scientist Jill Bolte Taylor recounted: *I could no longer define the boundaries of my body. I can’t define where I begin and where I end, because the atoms and molecules of my arm blend with the atoms and molecules of the wall, and all I could detect was this energy… I was immediately captivated by the magnificence of the energy around me. And because I could no longer identify the boundaries of my body, I felt enormous and expansive. I felt at one with all the energy that was, and it was beautiful*^1^. Recounting the sensations that arose after seeing the Earth from space, astronaut Edgar Mitchell said: *And suddenly I realized that the molecules of my body, the spacecraft, the body of my partners, were all manufactured in some ancient generation of stars. I felt an overwhelming sense of oneness, or connectedness… an insight, an epiphany*^2^*.* During a particularly potent meditation session Zen master Sokei-an Sasaki described how: *I lost the boundary of my physical body. I had my skin, of course, but I felt I was standing in the center of the cosmos… I saw people coming towards me, but all were… myself!*^3^ Recounting the sensations which arose from a high dose of the psychedelic drug (YD) psilocybin, a participant in a 2008 Griffiths et al. study recounted a *feeling of no boundaries, where I didn’t know where I ended and where my surroundings began. Somehow I was able to comprehend what oneness is*^4^.

These accounts, which express a profound sense of unity with other beings and other objects, stand in contrast to our typical day-to-day perspective, in which we often default to conceptual representations of ourselves (and others) as separate objects rather than coupled interdependent subjects. Yaden et al. have coined the term ‘self-transcendent experiences’ (STEs) to describe transient mental states in which *the subjective sense of one’s self as an isolated entity can temporarily fade into an experience of unity with other people or one’s surroundings, involving the dissolution of boundaries between the sense of self and ‘other’*^5^. Research across psychology, neuroscience, philosophy, pharmacology, and theology has drawn attention to the meaning and insight attributed to STEs by those who undergo them. Because they have been studied across so many different knowledge domains, they are described using different terminology, conceptual frameworks, and theoretical lenses, which makes it complicated to perform a comprehensive analysis of their subjective qualities, behavioral effects, and therapeutic potential. In an attempt to draw conceptual links across these various domains, Yaden et al. have identified a number of states as STEs, including for example F﻿low states^6^; Mindfulness states^7^; Awe^8^; Peak Experiences^9^; and Mystical-Type Experiences^10^.

**Figure 1:**
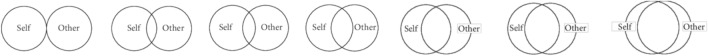
The Inclusion of Other in the Self Scale, which offers a continuum for expressing the sense of unitive feelings.

The phenomenology associated with different classes of STEs varies in saliency and visceral potency. As such, STEs have a broad range of intensities, some of which are more memorable, and thereby more significant for the person undergoing the experience. For example, “losing oneself” whilst reading a book or writing computer code may represent a relatively weak STE; whereas the deep sense of interdependence with the cosmos that arises from a spiritual ‘God-encounter’ experience represents a considerably more intense form of STE^11^. Analysis by Yaden et al. suggests that STEs involve at least two interrelated phenomenological ingredients: (1) an ‘annihilation’ of the sense of self, accomplished by dissolving self-boundaries and self-salience; and (2) a ‘relational’ sense of unity with something beyond the self (e.g., others or the natural world). The intensity of an STE is associated with the degree to which the participant experiences these interrelated phenomenological ingredients. While the precise sense of ‘self’ is difficult to define here, several different studies have nevertheless demonstrated the effectiveness of the so-called Inclusion of Other in the Self (IOS) Scale^12^ for assessing the degree to which an individual feels a sense of connectedness to others or to their environment. As shown in Fig. 1, this scale represents individuals and others as distinct circles. During weak unitive experiences, overlap between the circles is small; during particularly intense unitive experiences the overlap is more significant, representing an individual’s sense that ‘the other’ forms a significant part of the self.

William James suggested that particularly intense classes of STEs—namely ‘mystical type experiences’ (MTEs), where participants often describe a profound and ineffable sense of internal and external unity, changes in the experience of space and time, and interwoven senses of connectedness, sacredness, and noetic qualities—can have lasting positive, transformative, and life-changing effects. Recent analysis of questionnaire data from more than 4000 participants by Griffiths et al.^11^ seems aligned with James’ supposition: individuals who had undergone intense MTEs arising from subjective spiritual experiences rated them to be amongst their most meaningful and spiritually significant life experiences, associated with persistent positive changes in life satisfaction and purpose. Given their long-term therapeutic benefit, increasing research effort has been directed to explore technologies for reliably eliciting MTEs in a laboratory context. In this respect, psychedelic drugs (YDs) have emerged as a particularly promising way of producing subjective phenomenology similar to non-drug MTEs^11^, and are associated with lasting therapeutic benefit in addressing depression, addiction, and end-of-life-anxiety^4,13–16^. Unitive experiences of interconnectedness are common during YD experiences, where they are often associated with subjective perception of ego dissolution^17–19^, near-death-like experiences^20^, altered perception of both time and space^21^, and a sense of the ineffable^11^. For example, a 2006 Griffiths et al. study showed that 67% of participants who had subjective MTEs while taking psilocybin as part of a ‘psychedelic psychotherapy’ program considered the experience to be amongst the most meaningful experiences of their lives. Despite their potential, YDs face a number of practical challenges to widespread administration. For example, they require extensive psychotherapy support because they produce intense phenomenology that sometimes lead to transient fear/panic and associated physiological responses^13,22^, the onset and duration of which is often outside the therapist’s control, making it difficult for them to positively direct a patient’s experience. Moreover, ongoing questions remain as to how to best determine a YD dose that reliably elicits therapeutic effects but minimizes the aforementioned risks. Finally, YDs face significant regulatory challenges, which vary considerably across cultures.

**Figure 2:**
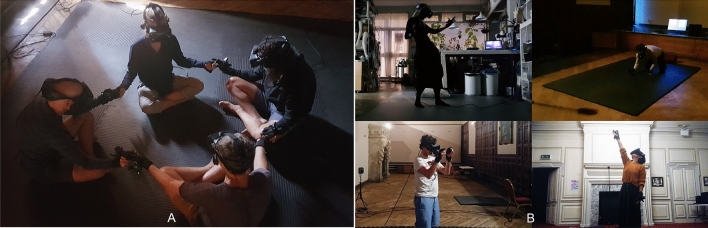
(**A**) the co-located version of Isness (Isness-C), an intersubjective multi-person group VR experience which Glowacki et al. showed produces MEQ30 scores comparable to moderate to high doses of YDs; (**B**) the distributed version of Isness (Isness-D), with four participants simultaneously joining a session from different Isness-D nodes distributed across the world.

Given the various challenges associated with YD administration, there has been emerging research exploring non-drug technologies for reliably eliciting STEs. Virtual Reality (VR) has emerged as a particularly interesting candidate^23,24^ given its ability to create strong alterations in perceptual phenomenology. For example, Glowacki et al.^25^ recently described a multi-person VR experience called ‘Isness’, where four participants co-located in the same physical and virtual space (Fig. 2A) experience the collective emergence, fluctuation, and dissipation of their bodies (and those of their co-participants) as diffuse energetic essences. The subjective MTEs produced by this co-located version of Isness (Isness-C) were evaluated (N = 57) using the MEQ30^26,27^, a commonly used YD experience questionnaire^28^. Glowacki et al. compared the Isness-C MEQ30 results to those obtained from more than 540 participants in 26 previous YD studies and showed that the Isness-C scores were statistically indistinguishable from MEQ30 scores following moderate-to-high doses of YDs. Isness-C participants reported positive emotions, a unitive sense of connectedness, a weakening of ego boundaries, and awareness of pure presence that was less distracted by ego. Isness-C offers a case study demonstrating that multi-person VR can be used to design group experiences that create the conditions for intense STEs from which participants derive insight and meaning. To distinguish technologies like Isness from psychedelic technologies, Glowacki et al. coined the term *numadelic*. Combining the Greek words *pneuma* (πνευμα, ‘breath’, ‘spirit’, or ‘soul’) and delein (δηλε*l*ν, ‘to reveal’, ‘to make visible’, or ‘to manifest’), *numadelic* may be translated as ‘spirit-manifesting’ or ‘spirit-revealing’. The *numadelic* aspect of Isness-C arises from the fact that participant bodies are represented as luminous energetic essences, an aesthetic representation associated with ‘spirit’ in a variety of wisdom and meditation traditions.

While Isness-C was (to the best of our knowledge) the first study using the MEQ30 to analyse subjective participant experience in a VR context, previous work has investigated the ability of both 2d and immersive video content to elicit two phenomenological ingredients known to be important components of a subjective YD-MTE, namely: (a) altered visual perception^29^, and (b) a sense of awe^23,30^. The former has been studied by Suzuki et al., who presented individuals with immersive 360 videos derived from Google’s deep dream convolutional neural nets, rendered in a VR headset^31,32^. Their results (N = 12) suggested that it is possible to induce visual phenomenology similar to psilocybin; however, they were unable to evoke in participants the corresponding sense of temporal distortion that psilocybin produces. The latter—awe—has been the topic of several studies. Broadly, these studies have shown that awe can be reliably elicited by presenting individuals with video content of various environments, e.g.: views from a mountaintop, within a vast open space, seeing earth from space, submerged in the ocean, amongst very tall trees, etc^33–38^. Recent fMRI studies suggest that participants presented with awe-eliciting 2d videos have reduced activity in the brain’s default mode network^39^, which is an ego-reaffirming neurological network that YDs also attenuate^15^. Chirico et al. showed that immersive videos rendered in head-mounted-displays (HMDs) are able to elicit a stronger sense of awe compared to standard 2d videos^33^, given that VR enables a heightened sense of immersion^40,41^.

The two items which Yaden et al. identified as primary phenomenological ingredients of STEs—(a) a weakened sense of self-boundaries and diminished self-salience; and (b) a ‘relational’ sense of unity with something beyond the self—are not easy to untangle. The difficulty becomes apparent by undertaking a simple thought experiment, wherein we imagine the dynamics that arise when two drops of liquid water coalesce: their individual identities and associated properties fade away as their respective boundaries co-mingle and eventually dissolve into one another, eventually creating a drop with new physical properties. Characterizing the softness, fluidity, or porosity of a body’s boundaries is impossible without corresponding observations of the dynamics that unfold when it undergoes some form of relational interaction with another body. The tight relational co-dependence of these phenomenological ingredients, in which one is required to characterize the other and vice-versa, suggests that they are perhaps *best realized in a multi-body, relational context*. This observation highlights a potentially significant gap in YD laboratory studies and efforts to design STEs using VR: *nearly all of the work described to date involves individual, intrasubjective experiences*.

## Present work

**Figure 3:**
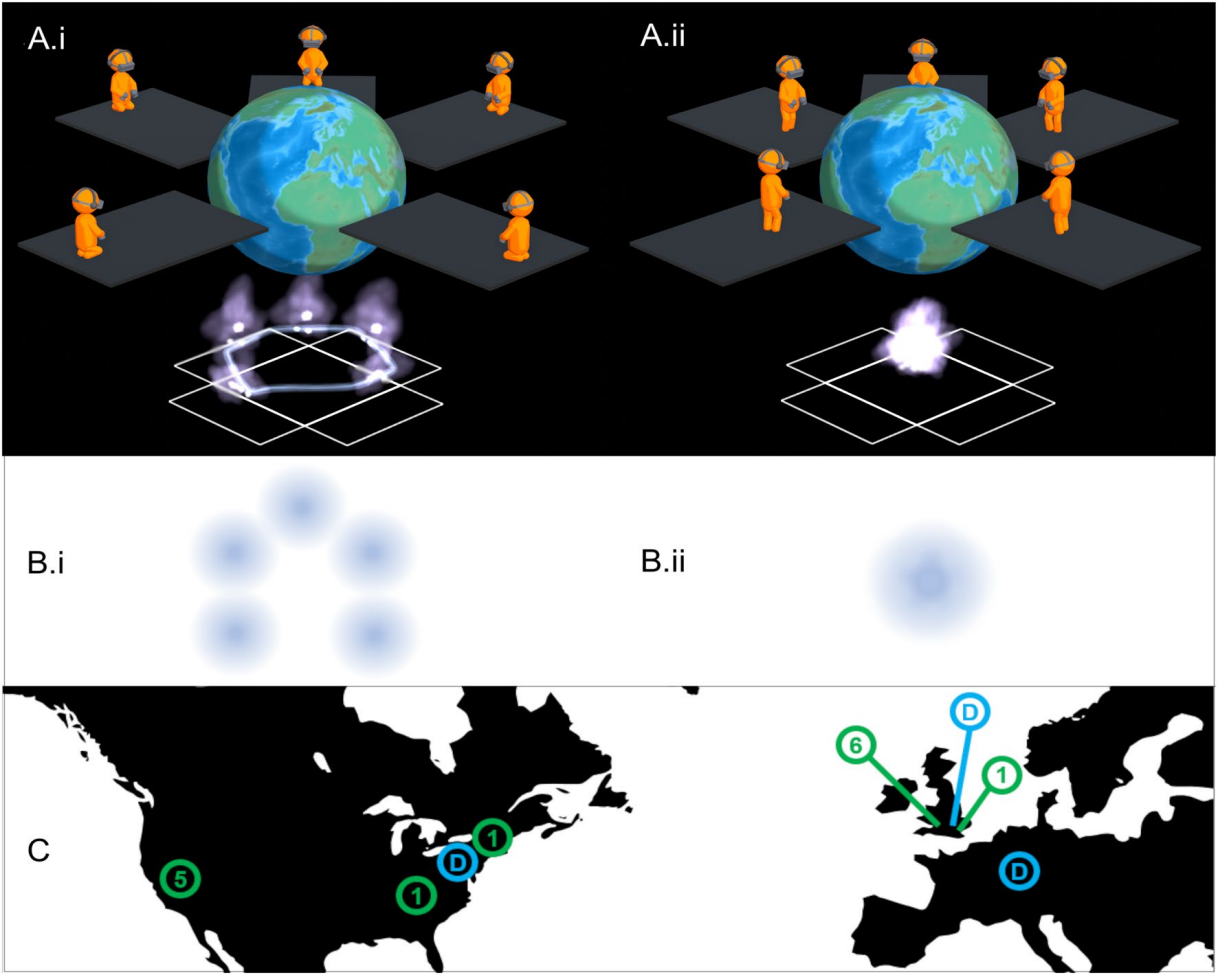
(**A.i**) shows 4 participants + 1 facilitator joining Isness from various ‘nodes’ distributed across the world. Participants are kneeling at the edge of their space and are represented in-world as energetic essences connected by a tangible dynamical molecular thread (which they ‘hold’ in their hands). (**A.ii**) shows participants undertaking ‘energetic coalescence’. Standing at the center of their respective mats, their energetic bodies overlap as they occupy the same position in the shared virtual space. The pattern on the floor in (**A.i**) and (**A.ii**) shows how participants’ local ‘play spaces’ were oriented within the virtual space. (**B.i**) & (**B.ii**) show IOS diagrams corresponding to (**A.i**) and (**A.ii**), for analogy with Fig. 1. (**C**) shows the international Isness ‘node’ locations as circular markers labelled with the total number of nodes in that location. There were 5 nodes in the San Francisco Bay Area (USA), 1 node in Tennessee (USA), 1 node in Massachusetts (USA), 6 nodes in Bristol (UK), and 1 node in London (UK). The Isness server was mounted on data centers D in London (UK), Frankfurt (Germany) and Washington D.C (USA).

In what follows, we describe our efforts designing intersubjective group STEs which enable participants distributed across the world (e.g., as shown in Figs. 2B and 3A) to collectively co-habit a shared virtual space and undergo an embodied experience of the overlap shown in the Inclusion of Other in the Self Scale in Fig. 1 (albeit in 4 dimensions (*x*, *y*, *z*, *t*) rather than 2 dimensions). Specifically, we constructed a multi-person cloud-mounted VR experience called Isness-distributed (Isness-D), which is illustrated in Fig. 3A,B. Harnessing the unique affordances of distributed multi-person VR, Isness-D’s *numadelic aesthetic* blurs conventional boundaries between self and other. Isness-D participants are rendered as luminous energetic essences with radiance concentrated at the heart centre. Compared to the hard boundaries illustrated in the IOS Scale (Fig. 1), Isness-D body boundaries are diffuse, fuzzy, and soft: they extend beyond the limits of the physical body, making it difficult to specify clearly where one body ends and another begins, as shown in Fig. 3B. This energetic aesthetic takes inspiration from subjective 1st person accounts of intense unitive experiences like those described at the beginning of this article, which often report a heightened sensitivity to a more fundamental energetic essence, and to the molecular and atomic constituents that combine to create our bodies and everything around us. The *numadelic* aesthetic is aligned with fundamental insights of modern physics (e.g., quantum mechanics), which emphasizes that objects have wave-like energetic essences and behaviors. Unlike their classical counterparts, waves are energetic processes and fundamentally non-local. This emphasis on matter’s *energetic* essence (rather than a localized materiality) is famously encapsulated by David Bohm’s description of matter as ‘frozen light’. Karl Popper similarly observed: *Matter turns out to be highly packed energy, transformable into other types of energy; and therefore something in the nature of a process… the results of modern physics suggest that we should give up the idea of a substance or essence… there is no self-identical entity persisting during all changes in time…*^42^.

The embodied experience of energetic overlap, illustrated in Fig. 3A,B—where participants can fluidly merge their energetic virtual bodies with those of the others in the group—we refer to as ‘energetic coalescence’. To the best of our knowledge, ‘energetic coalescence’ *represents a new class of experience which is only possible within a multi-person distributed VR environment—i.e., it cannot be realized in any other way*. This distinguishes Isness-D from previous work using VR to investigate STEs, which use VR to *simulate* STEs that are in principle available in the real world (YD visuals in the case of Suzuki, or awe experiences in the case of Chirico). In this respect, *‘energetic coalescence’* is similar to the ‘body-transfer’ work of Slater and co-workers (which enables a VR participant to inhabit the body of another person)—i.e., *a ‘body-transfer’ experience is only possible* using VR^43^. Whereas ‘body-transfer’ is an individual experience, ‘energetic coalescence’ is a group experience. The ‘energetic coalescence’ experience may represent a particularly explicit form of ‘identity fusion’, which ‘entails a visceral feeling of oneness with the group’^44^. As an experience of intense togetherness that temporarily transcends social structures, dissolving the norms that typically characterize relationships within structured institutional contexts, ‘energetic coalescence’ may represent an example of what Victor Turner called ‘communitas’^45^.

Isness-D is a group VR experience in which participants experience the emergence, dissipation, and fluctuation of their own bodies and the body of a simulated molecular ‘organism’ as energetic essences. Participants are guided by a combination of a live in-world facilitator and a pre-recorded narrative focused on the matter-energy relationship (which is described in further detail in the “Methods” (Sects. 6.8–6.9)). The software used to run Isness (described previously in ref^25^) grew out of a multi-person room-scale VR framework^46,47^ we initially designed to enable individuals^47–49^ and then groups^47,50–52^ to cohabit virtual environments where they can reach out and sculpt the dynamics of simulated molecular objects^47–49^. Our work over the years suggests that people interacting with these simulations (who from the outside appear to be doing little more than waving their hands around in the air) are able to ‘feel’ differences in the simulated dynamical flexibility of different molecular objects^47,53^. This highlights a crucial affordance of VR: *a modality that enables a tangible sensory experience of touching phenomena whose essences are purely energetic*. For designing STEs, this is a particularly useful affordance, given that it is aligned with subjective YD accounts where participants report sensitivity to the energetic nature of their environment—famously encapsulated by Aldous Huxley’s account of his mescaline experience. In a vase of flowers, he reported seeing “*the miracle, moment by moment, of naked existence… flowers shining with their own inner light and all but quivering under the pressure of the significance with which they were charged… [even] the folds of my grey flannel trousers were charged with is-ness.*”^54^ The presence of a molecular object whose dynamics were simulated in real-time was therefore an important intersubjective aspect of Isness-D. It represented for participants a kind of tangible, fluctuating organism (referred to at various points in the narrative as a ‘molecular organism’ or ‘energetic thread’) with a purely energetic essence, inviting people to recognize that their own essential energetic quality is shared with every other object (living and non-living) in the natural world. The depth of the Isness-D matter-energy narrative was reinforced by the fact that the dynamics of the molecular object were calculated in real-time using a state-of-the-art GPU-accelerated computational biophysics engine^55^. This sophistication anchored the Isness-D narrative in physical and scientific reality, encouraging participants to reflect on the fact that everyday material objects *are actually* constructed from the dynamical choreography of molecular organisms whose essences are fundamentally energetic^49^, an insight which arises in many first-person STE accounts (e.g., those at the beginning of this article). As shown in Fig. 3A.i, *multiple participants rendered as energy bodies, connected across different locales and time zones via an energetic molecular thread, represents a class of experience that is only possible using the affordances of multi-person distributed VR.*

Given that our laboratory (like many labs) was shut down as a result of COVID restrictions, we evaluated Isness-D using a ‘citizen science’ approach, coordinating a set of 14 “Isness-nodes” distributed across the world (Fig. 3C). Each node operated as a portal where a single participant could virtually enter into the shared Isness-D experience along with participants from other nodes. To analyse Isness-D, we again utilized the MEQ30 in order to facilitate direct comparisons with Isness-C. To assess other phenomenological ingredients known to be important during intense STEs, we used three additional measurement scales. First, we measured the extent to which participants in Isness-D experienced a unitive sense of connectedness with those joining from other Isness-D nodes. To measure this, we used an adapted version of the Inclusion of Community in Self (ICS) Scale, which is essentially a 5-item version of the IOS scale shown in Fig. 1, where participants were asked to rate the extent to which a circle representing the community of ‘Other Isness Participants’ overlapped with their sense of self^56^. Second, we measured the extent to which participants experienced a diminished sense of egoic identity, which (as discussed above) is closely interrelated with the intensity of the unitive experience, prominent within MTEs and YD experiences. To do this, we utilized the ego-dissolution inventory (EDI), which measures aspects of both ego dissolution and ego inflation, and has been utilized in prior YD studies^57–60^. Third, given that Isness-D was constructed as an intersubjective group experience, we attempted to measure the extent of ‘communitas’, which has been defined as an experience of intense togetherness and shared humanity that temporarily transcends social structures, mediated by an anti-structural and often ritualized ‘liminal’ phase of equality amongst community members^45^. To do this, we used the recently validated psychedelic communitas scale from Kettner et al.^61^ who showed that communitas experienced during intersubjective group YD experiences predicts enduring changes in psychological well-being and connectedness, with associated improvements in wellbeing, depressive symptoms, trait anxiety, and interpersonal tolerance.

Isness-D offers an interesting case study in developing technologies, methods, strategies, and protocols to carry out distributed multi-person studies in scenarios where face-to-face laboratory studies and clinical interactions are difficult. The lockdowns, social distancing, and uncertainty that have arisen as a result of the public response to the COVID-19 pandemic have created the conditions for increased social isolation and loneliness, which are correlated with feelings of fear, anxiety, stress, depressive thoughts, and helplessness^62–64^. While the longer term effects of COVID remain to be determined, past studies have highlighted social disconnectedness to be a key feature of depression^65^, while feelings of connectedness are associated with reduced depressive symptoms^66^. By encouraging participants to imagine their own bodies and those of others as energetic essences whose luminosity extends beyond the boundaries of their material form, Isness-D offers participants new experiences of connectedness, which may help address the isolation resulting from COVID. In this respect, experiences like Isness-D respond to recent calls to use human–computer interaction (HCI) technologies to design meaningful experiences^67^ which deal with the fundamentals of human existence (e.g., mortality, identity, isolation, meaning)^68,69^ in order to stimulate alternative narratives and visions within interconnected systems^70,71^ and explore new forms of spiritual sensitivity^72,73^.

## Results

### Sample size, demographics, and emotional response

From Aug–Sept 2020, we carried out 29 Isness sessions distributed across our network of 14 Isness nodes, with 109 total participants. 24 participants were not included in the analysis because their participation formed part of a technical training session or support role (i.e., as citizen science ‘volunteers’). Nine participants were removed from the analysis because the facilitator or node host identified that their experience suffered significant technical difficulties—primarily glitches and dropouts as a result of network instability. This left 75 remaining participants whose Isness-D sessions could be analyzed. Of these, 54 answered the pre-session questionnaire, and 58 answered the post-session questionnaire. Of these 58 participants, 59% were male, 37% were female, 2% were other, and 2% declined to state. Ages ranged from 23 to 75 years old with a mean (SD) of 38 (10) years. Over half the participants (57%) had not been in VR at all over the last year. Four (7%) had used VR 20 + times in the last year. 12% had experienced the earlier, co-located version of Isness^25^. One reported a brief period of nausea (they did not comment on the strength or duration and finished their session as usual). Three reported body aches and three reported headaches. Six participants reported crying at some point during their Isness-D experience. Anecdotally, our study team obtained several other reports of people who experienced a brief period of tears. One participant reported trembling and two felt sweats or chills. None of the participants reported these emotional reactions to be negative or traumatic.

### MEQ30

**Figure 4:**
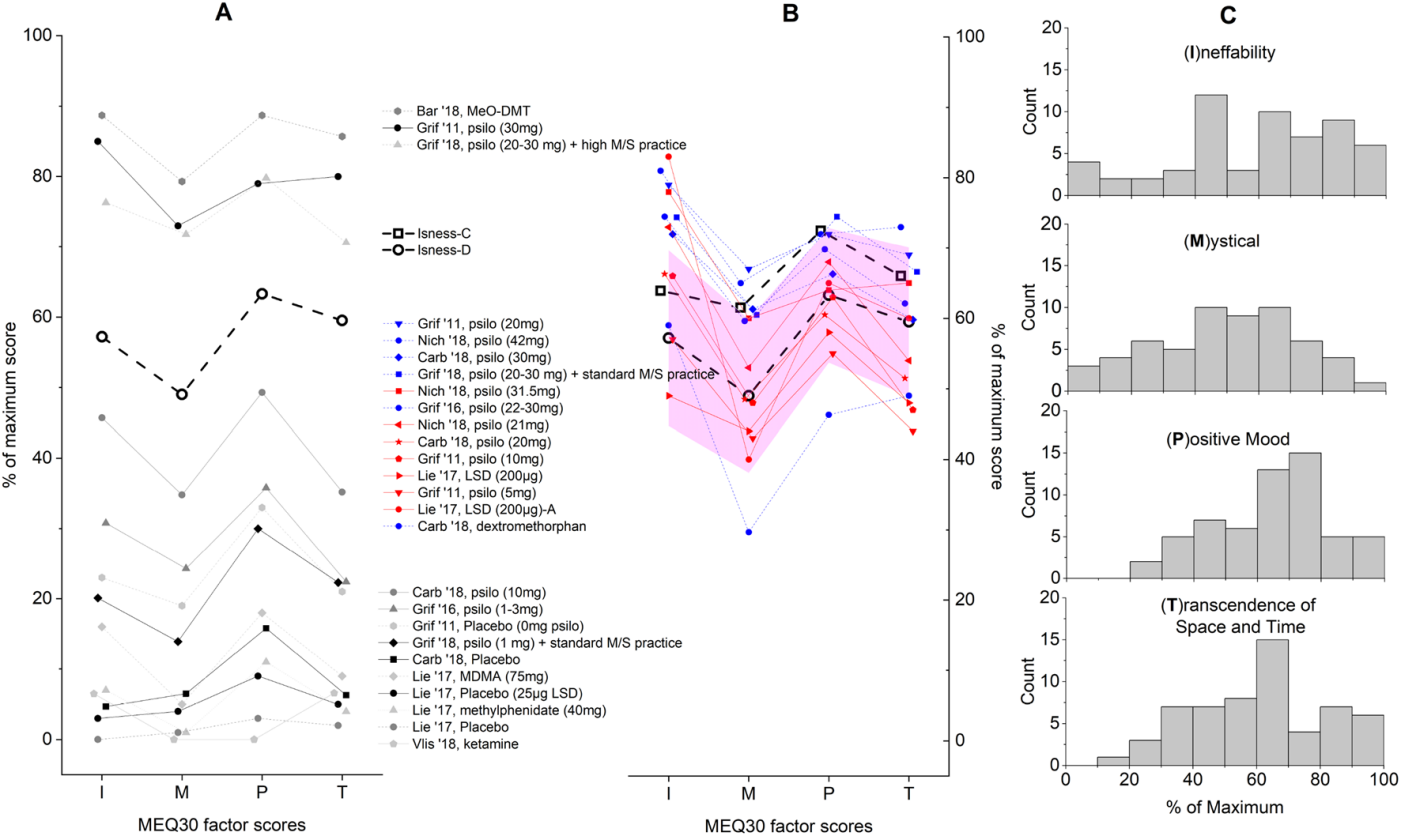
Comparison of the average (**I**, **M**, **P**, **T**) factor scores of Isness-D to Isness-C^25^ and previously published research studies that have employed the MEQ30 (Bar ’18^74^, Carb ’18^77^, Griff ’11^14^, Grif ’16^79^, Grif ’18^75^, Lie ’17^78^, Nich ’18^76^, Vlis ’18^81^, and Isness-C^25^). (**A**) shows the average factor scores for the studies that were statistically distinguishable from Isness-D (p < 0.05). (**B**) shows studies that were statistically indistinguishable from Isness-D on at least 2 factors (p > 0.05), where the blue lines indicate a study with two factor scores indistinguishable from Isness-D, and the red lines indicate a study with three or four factor scores indistinguishable from Isness-D. The area highlighted in pink shows the approximate region within which a factor score was determined to be indistinguishable at a 5% confidence level. (**C**) shows the distribution of the four factor scores of the MEQ30 results of Isness-D.

The MEQ30^27^ is a questionnaire that can distinguish dose dependent effects of YDs. It asks participants to rate the intensity with which they experienced 30 items on a 6-point scale [from “0 = none; not at all” to “5 = extreme (more than ever before in my life and stronger than 4)”], with three questions to capture ineffability **I**, fifteen mystical **M** [capturing unitive experiences, noetic quality, and sacredness], six positive mood **P**, and six transcendence of time/space **T**. Participant responses for each factor (**I**, **M**, **P**, **T**) are then averaged, and reported as a percentage of the maximum score. Figure 4 compares the Isness-D MEQ30 factor scores to 27 previous studies: 26 studies in the altered states database^28^, where the MEQ30 has been used to analyse YD altered states, and our previous Isness-C study^25^. To analyze these results, we followed the approach Barsuglia et al.^74^ used to analyze MEQ30 results obtained during 5-MeO-DMT field tests. Specifically, we undertook comparative analysis of Isness-D to the previously published studies using independent sample t-tests with a significance level of 5% (α = 0.05) calculated using Python (see SM for further details). Our previous work has shown that independent sample t-tests give results broadly aligned with more sophisticated statistical analyses^25^. Figure 4 and Table SM2 show the results of 27 different independent-sample t-tests, comparing the Isness MEQ30 results to each of the studies in Table SM2. To make Fig. 4, we analyzed whether a study was statistically indistinguishable (p > 0.05) from Isness-D on each of its **I**, **M**, **P** or **T** factor scores^25^. Compared to Isness-D, Fig. 4 shows:

- **3 studies which were more intense on all 4 factors**: (1) a 5-MeO-DMT study^74^; (2) a 30 mg psilocybin study^26^; and (3) a 2018 study by Griffiths et al.^75^ where participants in a program offering high levels of support in carrying out meditation and spiritual (M/S) practice were given 20–30 mg psilocybin
- **6 studies which were more intense on 2 factors**: Five of these studies involved participants being administered doses between 20–42 mg of psilocybin. The sixth included our previous Isness-C study^25^.
- **7 studies of equal intensity (indistinguishable on 3 or 4 factors)**: 3 studies were indistinguishable on all 4 factors (a 21 μg psilo study^76^, a 20 mg psilo study^77^, and a 200 μg LSD study^78^), and 4 were indistinguishable on three factors (psilocybin studies of 5 mg^14^, 10 mg^14^, 31.5 mg^76^, and a 200 μg LSD study^78^).
- **1 study which was less intense on 2 factors**, where participants were given dextromethorphan^77^.
- **10 studies which were less intense on 3 or 4 factors**: Amongst these, 6 were baseline studies, and 4 were drug-administration studies. The baseline studies included: 4 placebo studies; a 2016 study where Griffiths et al. gave participants sub-perceptual (1–3 mg) doses of psilocybin^79^; and a 2018 study where Griffiths et al.^75^ gave 1 mg psilocybin to participants in a program offering M/S practice support. The drug-administration studies investigated: MDMA^78,80^; methylphenidate^78,80^; ketamine^81^; and psilocybin^14,77^.

Griffiths and co-workers identify an MEQ30 respondent as having had a ‘complete MTE’ when each of the **I**, **M**, **P**, **T** factor scores are ≥ 60% of the maximum^22^. In general, the fraction of participants reporting a complete MTE is proportional to the YD dose. Barrett and Griffiths^82^ reported a meta-analysis of high dose (30 mg/70 kg) psilocybin studies in 119 healthy volunteers^13,14,75^, and observed that **57%** of participants had ‘complete’ MTEs. Our analysis shows that **29**% of Isness-D participants had a complete MTE, compared to the previous value of **44%** for Isness-C.

### Inclusion of community in self scale

**Figure 5:**
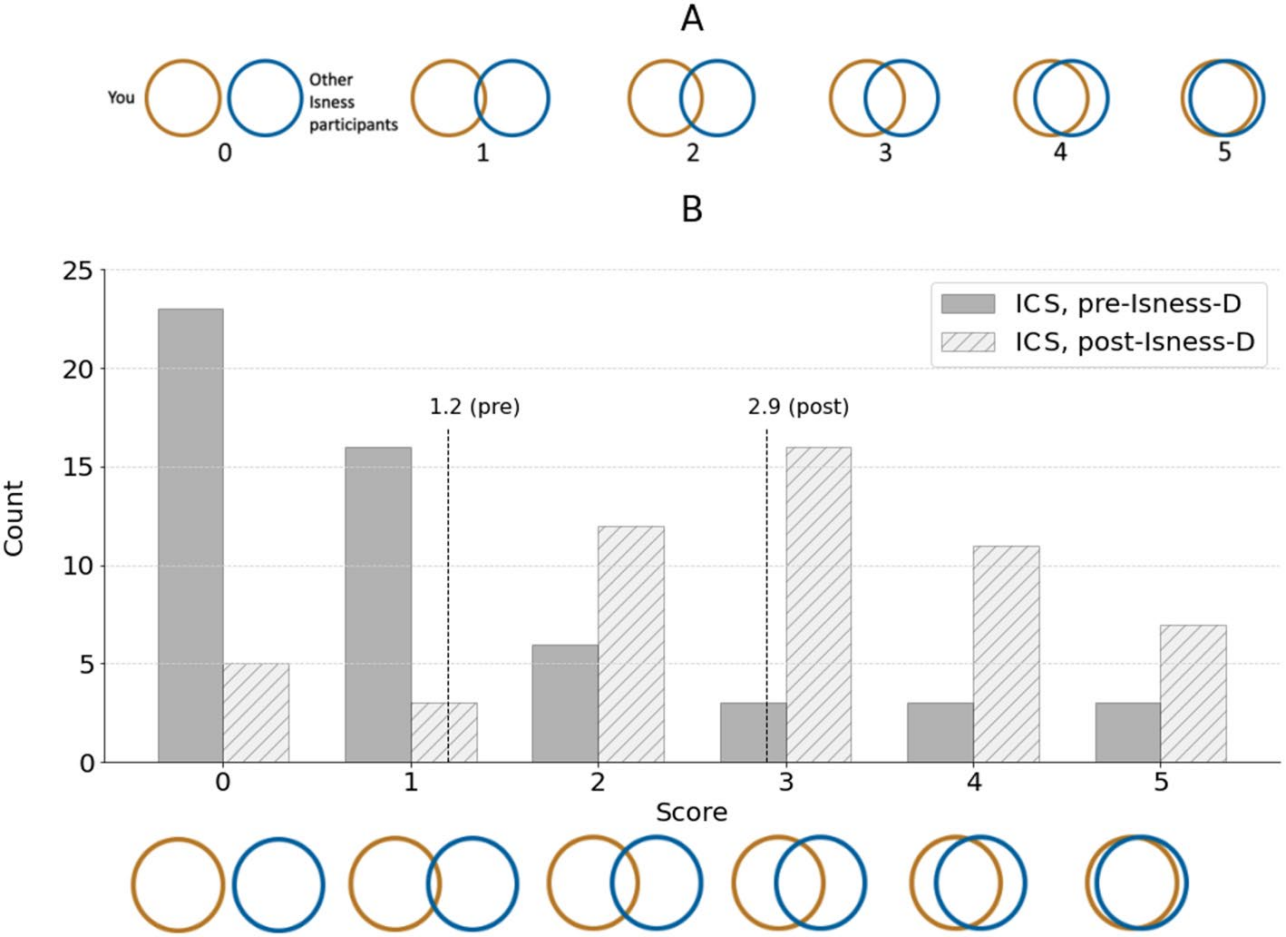
Panel (**A**) shows the ICS scale shown to participants in the pre-Isness-D and post-Isness-D questionnaire. Each picture has been labelled numerically from 0 to 5 for analysis, however, the pictures in the questionnaires were labelled alphabetically from (a) to (f). Panel (**B**) gives histograms of the ICS scores for all participants before and after Isness-D, with dashed lines indicating the respective averages.

54 participants answered both the pre and post questionnaires, both of which included the aforementioned ICS scale shown in Fig. 5. The ICS asked which set of circles best describes their relationship with the other Isness participants from a series of images. The first image showed two circles with no overlap (a score of 0) and the last image showed two circles almost entirely overlapping (a score of 5). Figure 5 shows a significant shift in the distribution of ICS scores following Isness-D, with participants indicating higher degrees of connectedness. A Wilcoxon signed rank test showed that the difference between the pre and post values was statistically significant (p < 1E-6), with the mean ICS rating of 1.2 ± 1.5 (SD) pre-Isness-D increasing to 2.9 ± 1.4 (SD) post-Isness-D. The post-Isness-D ICS results are statistically indistinguishable from recent results published by Forstmann et al*.*^83^ who investigated the effects of psychedelic substance use on positive mood and social connectedness of 450 participants (in naturalistic mass gathering settings) who used hallucinogens and/or euphorics in the last week. Given that the Forstmann et al*.*^83^ scale ranged from 1 to 7, we mapped their results onto a 0 to 5 scale to enable comparison. Forstmann et al. found an average IOS of 2.8 ± 1.3 (SD), which is statistically indistinguishable from the post-Isness-D results according to independent sample t-tests (p = 0.510).

### Ego-dissolution inventory (EDI)

**Figure 6:**
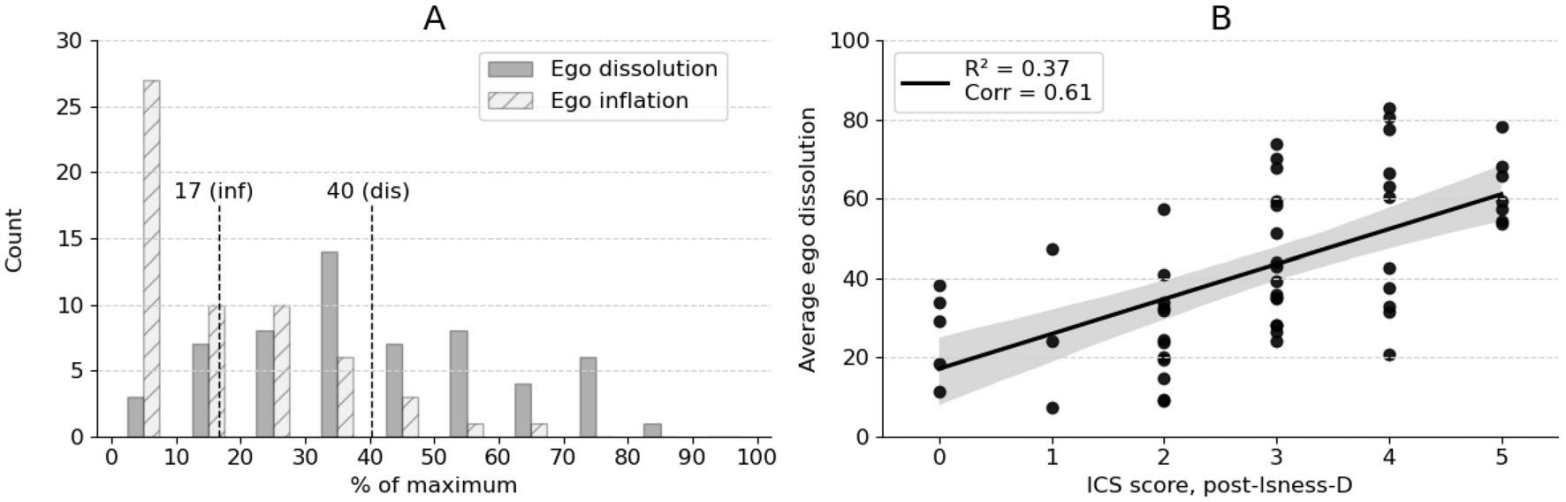
(**A**) distributions of the average scores for the ego inflation and ego dissolution items of the EDI across the Isness participants. The respective averages are indicated by the dashed lines. (**B**) scatter plot of the average ego dissolution against the post-Isness-D ICS scores for each participant, with a line of best fit calculated using a linear least-squares regression. The legend gives the Pearson correlation coefficient (‘corr’) and the R^2^ value. The corresponding p-value is 1.13 × 10^–6^.

The EDI^84^ self-report scale is typically employed in psychedelic studies to measure subjective experiences of ego dissolution. It consists of 16 items: 8 relate to ego dissolution (e.g., *I experienced a dissolution of my "self" or ego”, I felt a sense of union with others, *etc*.*) and 8 relate to ego inflation (e.g., *I felt more important or special than others*, *I felt especially sure-of-myself, *etc*.*). Following Isness-D, participants were asked to rate the extent to which each statement applied to their Isness-D experience on a scale of 0 (*no, not more than usually*) to 100 (*yes, I experienced this completely).* Cronbach’s alpha was calculated, and the results showed high internal consistency for both ego dissolution (α = 0.86) and inflation (α = 0.87). Figure 6 shows that the ego inflation distributions are shifted to lower values compared to the ego dissolution distributions, with a mean ego dissolution across the cohort of 40 ± 20 (SD), and mean ego inflation of 17 ± 15 (SD). The results in Fig. 6 are consistent with subjective participant reports following LSD-equivalent doses of ~ 100 μg, based on inspection of values published by Nour et al.^84^ Direct comparison of the complete Isness-D EDI scores to previous YD studies is complicated for the following reasons: (a) several previous studies use only a single EDI item to measure ego dissolution (usually *I experienced a dissolution of my “self” or ego*, or similar); and (b) for some of the previous studies where EDI was reported, patients were placed in an fMRI^57,58^ or PET scanner^59^, which may lead to anxiety and impact the reported scores^85^. For Isness-D, the mean for this specific question was 43 ± 29 (SD), which is higher (p < 0.001) than the subjective responses following 75 µg doses of LSD published by Tagliazucchi et al.^57^ and lower (p < 1E-14) than responses following 100 µg of LSD, published by Holze et al. (83 ± 10.2)^58^. In the same study, Holze et al. reported the mean ego dissolution across participants following a 125 mg dose of MDMA to be 44 ± 7.9 (SD), which was indistinguishable (p = 0.702) from Isness-D. Madsen et al.^59^ used the EDI to measure ego dissolution for participants following a range of doses of psilocybin (0–30 mg). Compared to Isness-D, Madsen et al. found similar or lower mean ego dissolution values following doses up to and including 18 mg of psilocybin. Increasing the psilocybin dose further led to higher mean scores for all participants compared to Isness-D. Mason et al.^60^ measured the subjective ego dissolution of participants following 0.17 mg of psilocybin per kg of body weight (avg ~ 12 mg dose per person) using the EDI. The mean across the participants was 30.7 ± 4.6 (SD), which is lower (p = 0.003) than Isness-D.

To evaluate the relationship between a participant’s sense of ego dissolution and their sense of connectedness, Fig. 6B shows a correlation plot of each participant’s average post-Isness-D ego dissolution against the corresponding post-Isness-D ICS score. While there is some scatter in the data, a linear least-squares regression indicated a positive correlation (R^2^ = 0.37) between the average post-Isness-D EDI score and the ICS score. The Pearson correlation coefficient (0.61) similarly indicated a positive correlation. These results are distinct from the weaker correlations observed between the post-Isness-D ego dissolution scores and the pre-Isness-D ICS scores (R^2^ = 0.17 and a Pearson correlation coefficient of 0.41). The change in correlation suggests that the sense of social connectedness which Isness-D elicits for participants correlates with a sense of ego attenuation.

### Communitas

**Figure 7:**
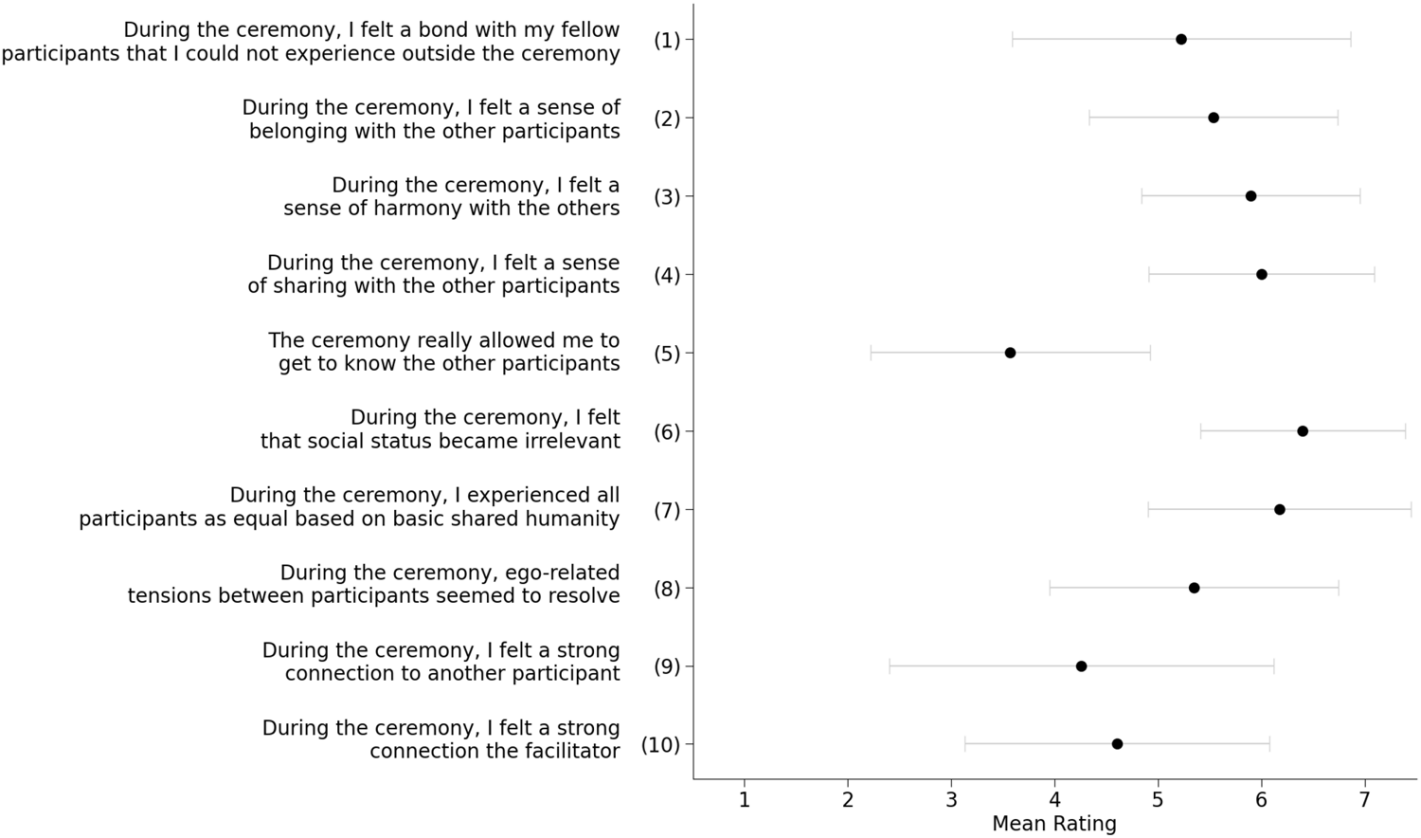
Mean ratings for the 8 items in the Communitas questionnaire (items 1–8), and two additional items relating to connection with another participant and with the facilitator (items 9 & 10), with ± 1 SD error bars.

Following Kettner et al.^61^ we used the scale in Fig. 7 to assess participants’ Isness-D experiences. The first 8 items offer a subjective assessment of communitas, item 9 assesses participant-to-participant connection, and item 10 assesses participant-to-facilitator connection. The scale ranges from 1 (*strongly disagree*) to 7 (*strongly agree*). Calculation of Cronbach’s coefficient showed high internal consistency within the 10 items (α = 0.84). A one-sample t-test against the midpoint of the scale showed that the overall mean of the 10 items across the participants was higher than the midpoint of the scale value (p < 0.001), indicating that a sense of togetherness was felt by participants during their shared Isness-D sessions. The item *the ceremony really allowed me to get to know the other participants* was the only one to receive a lower mean score than the midpoint scale value, perhaps because the Isness-D experience involved little verbal communication between participants. The rest of the items all received ratings higher than scale midpoint value, with the item *during the ceremony, I felt that social status became irrelevant* receiving the highest mean rating. In recently published studies where Kettner et al. explored *collective* psychedelic use during YD ceremonies in naturalistic settings (N = 886), they calculated the mean reported total communitas as the sum across the first 8 items in Fig. 7. They obtained values of 39.58 (SD 11.23), corresponding to ~ 71% of the maximum score of 56. Isness-D produced mean reported communitas values that are statistically distinguishable (p = 0.002), with an average total communitas of 44.14 (SD 6.87), which corresponds to ~ 78% of the maximum score.

### Qualitative analysis

Our qualitative analysis is based on data from three sources: (1) semi-structured group discussion in VR during phase 3 of Isness-D; (2) optional free writing upon exiting VR; and (3) a post-Isness-D questionnaire. All the group discussions were recorded (~ 3.5 h total) and then transcribed (~ 14,000 words). We received 36 free writing responses in a variety of forms: prose (27 legible and 1 illegible), poetry (4 responses), and drawings (4 responses). Only the prose (~ 3000 words) was included in the thematic analysis. An inductive thematic analysis^86^ was used to categorize the qualitative data into themes. Any statements that involved multiple themes were assigned to the most relevant. The identified themes and the number of times each theme was observed are as follows: Connectedness^75^; Positive Emotions^65^; Embodied awareness^40^; Ego Dissolution^31^; Supportive Setting^28^; Sense of Play^24^; Transcendence of Space/Time^13^; Noetic Quality^11^; Comparison to other Altered States^10^; Sense of Beauty^9^; and Reflection on Mortality^8^. Table SM6 contains indicative quotations for each theme; the SM includes the entire thematic analysis classification. Several of the participant statements (*written in italics*) are woven into the discussion that follows.

## Discussion

Our results overwhelmingly suggest that the phenomenological intensity of STEs which arose for participants during Isness-D is comparable to YDs, in both naturalistic and laboratory settings. For example, Isness-D participants’ average ICS score of 2.9 ± 1.4 (SD) is statistically indistinguishable from recent results published by Forstmann et al.^83^ who found values of 2.8 ± 1.3 (SD) in a large-scale naturalistic study investigating YD effects for more than 450 participants. Similarly, the extent of communitas reported by Isness-D participants (44.14 ± 6.87, corresponding to ~ 78% of the max score) is statistically higher than the value of 39.58 ± 11.23 (~ 71% of the max score) reported in Kettner et al.’s recent N = 886 study investigating *collective* psychedelic use during YD ceremonies in naturalistic settings. Our EDI analysis indicates that the Isness-D results are comparable to YD drug does of ~ 18 mg psilocybin, 75–100 μg LSD, or 125 mg MDMA. These results are broadly consistent with the results of the MEQ30 analysis, showing 3 previous YD studies which were indistinguishable from Isness-D on all 4 factors: psilocybin studies of 20 mg^77^ and 21 mg^76^, and an LSD study of 200 μg^78^. The qualitative analysis indicates some phenomenological similarity between Isness-D and psychedelics, with participants for example observing how it was “*similar to experiences that I have had as somatic visions through medicine plants. The interconnective nature of energy/intention and the ‘strings’ that appear to interconnect us with all living matter [is] also related to childhood dreams I had prior to any ‘psychedelic experience’* ”. Others stated how Isness-D left them with a *“sense of interconnectedness… only previously noticed with the help of psychedelics in the right setting*”. Some commented on the emotional impact of Isness-D, “*I was amazed [by] how moved I was. I think it was the juxtaposition of the frustration and then the beholding beauty. I think the music really helped. It felt very beautiful to sit back and witness. I don’t normally get moved quite so much.”* Similar to YD experiences, some participants attributed a spiritual significance to Isness-D, “*That’s definitely a spiritual experience of some sort. It’s like, tangible*”. Because Isness-D grounds emergent spiritual concepts in rigorous physics insights like the fundamental relationship between matter and energy, it offers a case study for how careful human computer interaction (HCI) design can be used to cultivate spiritual sensitivities which avoid the “woo-woo” pseudo-scientific associations which can arise from YDs, e.g., which have recently been discussed by Carhartt-Harris and Friston^15^.

Connectedness, which has been previously been highlighted as a key aspect of the YD experience^87^, emerged as the strongest qualitative theme for Isness-D participants, “*I felt connected with myself but also with everyone else here… I think ‘connected’ is the word for me for the end of this session.*” Others commented on how Isness-D offered “*A completely other way of connecting that I’m not familiar with, [where] all the usual stuff disappears.*” Comments like these are aligned with the observed change in ICS post-Isness versus pre-Isness. The matter-energy narrative of Isness-D and its aesthetics of luminosity—which blurred the boundaries of individual identity—seems to have been important for enabling participants to reimagine a sense of connectedness: “*Connecting light and emotions as energy was special… light as connection; light transferring between matter; light creating memory. Experiencing myself and the other people in the group as light energy was joyful. It… allowed me to think about other spaces connecting in the world.*” While the experience of energetic coalescence was only one aspect of the Isness-D experience, it seemed to be particularly poignant and intimate for many people, with participants “*struck with how quickly the abstract lights grew to hold tangible meaning… as other people. It was especially poignant [during moments of coalescence] when we moved toward the centre and felt as if we might collide or enter each other’s personal space.”* For many participants, coalescence facilitated a particularly strong sense of connectedness: *“we could get closer than [in real-life] which felt more intimate, and connecting—nearly as much so as with a partner, child or pet—even though we were in different places.”* Others observed how moments of coalescence produced somatic sensations: “*I can just literally walk into people and it’s quite sensuous*”, and also “*I could feel subtle changes in my hands as if something was passing by, something physical*.” Another commented how, during moments of coalescence “*I got quite emotional… I got this surge of emotion where I don’t know if I wanted to gasp or cry or what it was, but I was kind of shocked in awe.”* Not a single participant reported being uncomfortable when others coalesced with their energetic essence; however, some worried they were invading others ‘personal space’: *“You can sense an imaginary presence around the glowing light, and you give it space even though there is no barrier there, other than imagined.”* Several commented on the sense of intimacy that accompanied coalescence “*It felt really intimate. And I tried to connect to whichever light being it was and… I tried to join mudras, and I think I sat [as an energetic essence] inside a few of you. I hope that was OK”* Many people reported a pleasant sense of spaciousness at the moment of coalescence, recognizing in that instant that conventional material boundaries did not apply, “*I found it really strange at the start, when you were telling us to go closer and closer and then that weird boundary of where personal space is and all of a sudden it was like ‘oh, actually this is kind of nice!’* ” Several participants commented on how the intimacy of the coalescence experience was balanced with a sense of innocence and purity, enabling “*the sweetest tenderness or pure, childlike love….*”

Earlier in this article, we highlighted two phenomenological ingredients known to be important in STEs: (1) diminished self-salience arising from dissolving self-boundaries; and (2) a sense of unity with something beyond the self. Given the tight relational co-dependence of these ingredients (where one is required to characterize the other and vice-versa), we suggested that they are *best realized in a multi-body context*, given that the softness, fluidity, or porosity of a body’s boundaries become most apparent by observing its relational interactions with another body. One participant succinctly articulated how they experienced this relational co-dependence during Isness-D: “*you lose yourself in the connection to other people*”. Figure 6B and Fig SM1 show that the correlation between participants’ sense of connectedness and their diminished sense of egoic identity was significantly stronger following Isness-D than it was beforehand: the diminished sense of ego entailed in “losing oneself” arises through the unique sort of “connection to other people” that Isness-D enables. The relational co-dependence between connectedness and diminished egoic identity is also seen in quotations like, ‘*Identity didn’t matter anymore; it was about experiencing things together. That was wonderful.*’ As energetic essences, the implicit, explicit, conscious, and unconscious judgments that permeate social and relational interactions were diminished: ‘*I found [the anonymity] quite powerful because we were all completely equal in the space. Any of the pre-judgements that come from how people look, sound and that sort of thing just aren’t there.*’ Stripping away these aspects of interpersonal interactions led to a sense of joy, purity, love, and beauty: ‘*I felt [when touching one another’s heart centres] the sweetest tenderness or pure, childlike love… stripped back, without any of the assumed layerings that we place upon reality and relationships… just to the absolute core, it was truly beautiful.’* These comments support the exceptionally high scores observed for item 6 in the Communitas scale in Fig. 7 (*During the ceremony, I felt that social status became irrelevant)*. Yaden et al.^5^ note that the experience of self-loss is sometimes linked to pathologies (e.g., going back to the work of Freud); however, they argue that it is more often associated with positive outcomes. Our results are broadly aligned with this conclusion: the experience of self-loss for Isness-D participants was overwhelmingly positive.

Participants reported positive emotions and an overwhelming sense of calm and relaxation at the end of their Isness-D experience, “*I feel remarkably happy. There’s something very happiness-inducing about this whole practice.*” Some reported lower levels of anxiety and stress, observing that Isness-D helped them to “*dissociate from something within you…I don’t know if it… was my identity or my anxieties that I put on the side…*” Another similarly commented, “*I arrived feeling pretty anxious and disconnected from others, but after that experience I feel much more calm and hopeful. Hugely due to the focus of how energy doesn’t truly disappear. I guess the fear of dying (the existential threat) is always on our minds, but that experience quieted the fear.”* For some, Isness-D encouraged reflection on mortality, and their own energetic transience amidst a larger unfolding energetic process: “*It’s clear that the energy isn’t just gone [in death], it goes somewhere. It’s beautiful to think that it’s all out there somewhere and it’s still circling you constantly*” and also “*Seeing all the lights together reminded me of a dream I had years ago where the universe was ending and everybody was dissolving into balls of light and merging into one. So, I was like ‘oh cool! The universe is ending. I’m cool with that.*’ ” Some participants articulated how Isness-D offered a form of connectedness that contrasts COVID-related isolation: “*[the sense of connection] is an experience I haven’t felt in a while because of COVID and being in front of screens all of the time”*, and also “*I feel like we’ve had a hug, and I haven’t had many of those recently… a really nice thing to have*”. Another commented on how it helped them understand the difference between “*stillness and stagnation…. This [pandemic] can make you feel very stagnant, cause you’re in the same place and you’re not still or rooted to anything.*” For many participants, positive emotions seemed to be associated with the sense of playfulness that arose from relaxed egoic identity: “*It was…a space to be free, you could do whatever you want and not feel judged”* and also “*There certainly was a sense of playfulness in this, which was really nice. As adults that’s not always something that we pursue.*”

For the MEQ30, the Isness-D and Isness-C results for the **I** and **T** factors were statistically indistinguishable. For the **M** and **P** factors, the Isness-D scores were lower than Isness-C. The lower scores make sense for several reasons. First, the distributed ‘citizen science’ approach meant that we had significantly less control over participant’s set and setting prior to entering VR and after leaving VR. Whereas in our previous study we were able to offer all participants the same context, the same preparatory environment, similar psychological priming, and a more consistent experience of the technology, this was not possible using the distributed citizen science approach, where the variability was more significant. For example, we had little ability to influence participant expectations or control how the node hosts described Isness-D to participants during the recruitment phase. Especially in the early stages, there was variability in host preparation: for example, there were some cases where participants from one node would be ready and waiting in VR for the beginning of Isness-D phase 1, whilst other node hosts were troubleshooting the technology (e.g., HMD fit, focus, fit of the OMG-VR gloves (see Methods), etc.). Second, whereas our previously published results were all obtained using a local area network where we could closely monitor latency and quickly solve any technical problems, the same was not true for the work described herein. During the early stages of our citizen science study, a number of participants had intermittent disruptions to their plausibility illusion^40,41^, as a result of unoptimized network instability issues. Third, whereas the previous participant cohort were drawn from attendees at a psychedelics & consciousness conference, the cohort for Isness-D was drawn from a broader distribution of international participants. Finally, Isness-D was effectively an adaptation of an experience that we originally designed to be co-located. Whilst we made several changes to accommodate the affordances of a multi-person distributed experience, our limited resources during the lockdown period made it difficult for us to undertake more extensive changes.

Isness-D differs from conventional YD psychotherapy in various ways. For example, the phase 1 preparation lasted ~ 15 min, far less than the preparation for studies carried out for YD psychotherapy, which typically include a total of 4–8 individual sessions (both before and after the YD session). The three phases of Isness-D last a total of ~ 70 min, shorter than psilocybin and LSD experiences, which can often last from 6–14 h. Finally, Isness-D was designed as a group experience, whereas most YD and VR studies are individual experiences. As discussed above, the distributed multi-body group aspect of Isness-D is clearly important for weakening ego boundaries, and fostering a sense of connectedness. The opening and closing interactions between the group and the facilitator (e.g., to develop the energy-matter narrative and encourage moments of energetic coalescence) were important, *‘really help[ing] to hold the experience and make me feel more comfortable with everyone.’* Compared to previous YD studies, the N = 58 Isness-D sample size is reasonable; however, this study had a number of limitations. For example, while we were able to demonstrate significant changes post-Isness compared to pre-Isness, we did not carry out a control experiment, and therefore some of our analyses are primarily comparative. Whilst the ‘citizen science’ participant sample in this work clearly represents an improvement on our previous work, further work will be required to make definitive statements about the extent to which sample selection bias may have influenced our results. Our ability to compare the MEQ30 from this work with previous studies depends on the assumption that the baseline responses of our participant sample are not anomalously high or low, and within the range spanned by 6 previously published baseline studies. Figure 4 shows that these baseline studies have a broad MEQ30 score distribution. Our comparative statistical analyses (Fig. 4 and Table SM2) show that the Isness-D results are more intense (p < 1E−6) than all of them. Nevertheless, we believe that the results described herein provide a degree of confidence in the results which we obtained in our previous study. As discussed above, the fact that the MEQ30 results for Isness-D are slightly lower than the results obtained for the co-located Isness-C experience is entirely aligned with our expectations, given the very different circumstances in which each study was conducted. At this stage, it is unclear how exactly to define a “placebo” for an experience like Isness-D, but this is an issue that would be interesting to investigate in future work. The design of Isness-D as a group experience suggests that the individual data may be correlated. In future studies we wish to investigate the correlation of the results obtained for participants within specific groups, and compare *intra*-group results to *inter*-group results. Given that the quantitative analysis was carried out retrospectively and not in relation to specific moments of Isness-D, we are somewhat limited in our ability to comment on how the narrative aspects of Isness may have primed participants for mystical-type, communitas and fusion experiences. Neither can we make conclusive statements regarding the extent to which the results in Figs. 4, 5, 6, 7 arise from specific components of Isness-D. In future studies, it would be interesting to address these limitations by carrying out control experiments in which aspects of Isness-D are systematically removed. This would enable us to understand in further detail the extent to which specific Isness-D components—the narrative, the immersive group aspect, the energetic coalescence, etc.—contribute to the overall results.

## Conclusions

The aim of the work described herein was to determine whether the unique affordances of multi-person distributed VR can be used to reliably elicit intense STEs. Within this paper, we have described Isness-D, an experience we have developed to blur conventional self-other boundaries using the unique affordances of distributed multi-person VR. Built on a matter-energy narrative, Isness-D enables groups of participants distributed across the world to co-habit a shared virtual space and collectively experience their bodies as luminous energetic essences with soft spatial boundaries. It encourages participants to imagine themselves, others, and the world around them as unfolding interconnected processes which are energetic (rather than fixed material entities). This fluid energetic representation enables participants to undergo moments of ‘energetic coalescence’, a new class of embodied intersubjective experience whereby participants can have an embodied experience of including multiple others within their self-representation. To evaluate Isness-D, we adopted a ‘citizen science’ approach, coordinating a network of nodes distributed around the world to run multiple Isness-D sessions. This strategy enabled us to carry out this research amidst COVID related social distancing constraints. As a distributed approach, it was difficult to prime Isness-D participants in a controlled way. Nevertheless, analysis of Isness-D participant scores on four different self-report scales commonly used to assess YD experiences overwhelmingly suggest that the phenomenological intensity of STEs which arose for participants during Isness-D is comparable to previously published YD studies, in both naturalistic and laboratory settings. Isness-D enables participants to dissolve their sense of self in the connection to others, relaxing attachment to egoic identity and facilitating a strong sense of connectedness. For many participants, Isness-D offered a sense of intimacy, innocence, playfulness, and purity, eliciting a state of calm spaciousness. To the best of our knowledge, this work represents the first attempt to analyze a distributed multi-person VR experience using measurement scales which are typically applied to YD experiences. These results demonstrate that distributed VR can be used to elicit intersubjective STEs which simultaneously attenuate egoic identity and facilitate a sense of connectedness. This study reaffirms the ideas in our previous work^25^, where we speculated that it is possible to design phenomenology and experiences using *numadelic* technologies like multi-person VR to create the conditions for STEs from which participants derive insight and meaning. Distributed intersubjective VR experiences like Isness-D may have a role to play in easing unprecedented feelings of loneliness, isolation, and fear that have arisen with COVID restrictions. In future work, we hope to explore in futher detail the mechanisms responsible for the results obtained during Isness-D, and also carry out detailed follow-ups with participants in order to understand its impacts over the longer term.

## Methods

### Ethics

Ethics approval for the study described herein was obtained from the Faculty of Science Research Ethics Committee at the University of Bristol (ethics approval code: 111003). Participants read an online study information sheet and completed a tick box consent form before signing up to an Isness-D session. The study was conducted according to the revised Declaration of Helsinki (2013) and the 1996 ICH Guidelines for Good Clinical Practice E6(R1). All participants were at least 18 years old, and consented to their data being gathered and published. To minimize participant risk, we adopted VR guidelines in line with those recommended by Madary and Metzinger^88^. All participants provided informed consent.

### Software & hardware

Isness-D is an approximately 50-min experience with conceptual roots in earlier work by Glowacki and co-workers^25,89–91^. As a fork of the Narupa project, Isness-D is designed around a client/server architecture enabling each VR client access to the positional data of all the other participants, and a shared real-time molecular simulation of a 40-Alanine peptide macrocycle (40-ALA) whose dynamics are calculated in real-time using OpenMM, a GPU-accelerated computational biophysics engine^55^. Each participant can see through their headset a visual representation of both the molecular simulation and all of the other participants (e.g., as in Fig. 3). We designed the Isness-D experience to accommodate 4–5 participants wearing either the HTC Vive Pro or Valve Index HMD. Our cloud computing resources enabled us to mount our server on GPU shapes available at three different data centres: Frankfurt (Germany), London (UK), and Ashburn (Maryland, USA). Prior to entering Isness-D, participants at each node were fitted with the Open Mudra Gloves for Virtual Reality (OMG-VRs), etextile gloves that sense when a user pinches together their thumb and index finger, or thumb and middle finger to form a "mudra" position^13^. The OMG-VRs were designed to enable research scientists and students to ‘grasp’ and ‘manipulate’ molecular objects whose essence is purely energetic. They provide good positional tracking of the point at which a pinch takes place, require no calibration, and proved sufficiently robust to withstand use across the international network of Isness-D nodes in Fig. 3C. The OMG-VRs played an important role during the Isness experience. By adopting a ‘mudra pose’ (bringing the tip of their thumb in contact with the tip of either their forefinger or middle finger), Isness-D participants could generate light and collectively sculpt the dynamics of the tangible molecular object. The in-world rendering of participants' bodies is designed so that the intensity of the light generated during moments of energetic coalescence depends on the overlap between energetic bodies. For example, two coalesced bodies generate more light than a single body, and four coalesced bodies generate more light than two.

### Isness nodes and citizen science design

Each of the 14 different nodes distributed across the world (Fig. 3C) was equipped with the equipment required to run Isness: (1) a room-scale play space of 2 m × 3 m; (2) a VR-capable computer; (3) an HTC Vive Pro or Valve Index HMD; (4) a single pair of OMG-VRs; and (5) a reliable internet connection. Each node was managed by a ‘host’: an individual whom we trained over Zoom/Slack to set up the VR hardware and to connect an Isness client into a cloud-mounted server instance. Hosts were given guidance on how to prepare participants prior to entering Isness-D (e.g. discussing what the experience would involve, creating a calming space, and offering participants an opportunity for quiet reflection), how to deal with participant discomfort, how to troubleshoot technology issues, and how to sanitize the VR equipment after participant use. In order to give hosts a sense of what their participants would experience during Isness-D, and also some indication of how the experience should feel, each of the hosts had the opportunity to undergo the full experience prior to engaging participants.

### Participants & data gathering

Participants for Isness-D were recruited by each of the node hosts. After the node hosts collectively agreed a schedule of Isness-D time slots during which they were available to accommodate participants, this list of timeslots was placed into a web scheduler, with a link that was then circulated to participants. After receiving the web link, participants were able to identify the various session times available at their host node. Before registering for an Isness-D time slot, they first had to read the online study information sheet and complete a tick box consent form. We based our data collection on the structure of the psychedelic ceremony study^92^, which involves a ‘pre-ceremony’ and a ‘post-ceremony’ component and includes a combination of psychometrics commonly used in psychedelic research studies. The online pre-Isness-D questionnaire (using the survey platform Qualtrics) consisted of the ICS scale^56^ and items relating to participants’ openness to the upcoming experience. The post-Isness-D questionnaire included: (1) the ICS scale^56^; 2) the MEQ30^26,27^ 3) the EDI^84^; and 4) the Communitas questionnaire^61^. Additionally, participants were asked questions related to bodily effects and demographics. We added to the post questionnaire a *final comments* section where participants were able to leave written feedback. Since it was designed to evaluate YD experiences, not all aspects of these pre- and post-ceremony questionnaires were relevant to Isness-D. To ensure its relevance, we undertook a number of modifications. For example, we removed any questions asking about *suffering* and *surrendering*; we judged these to be less relevant to the Isness-D experience and moreover our preliminary analysis suggested that they increased anxiety among participants. The final versions of the pre- and post-Isness-D questionnaires used to evaluate Isness-D are given in the SM. Participants received an email link to a digital version of the pre-Isness-D questionaire approximately one hour prior to entering into Isness-D, and another link to the post-Isness-D questionaire after the conclusion of their session.

### Isness-D experience design

Participants experienced Isness-D as a 3-phase journey, composed of a preparatory/induction phase, the Isness-D session, and an integration/reflection phase, adopting a similar model to that which we have utilized previously, and which is based on the model utilized in YD assisted psychotherapy^22^. An Isness-D session involved a facilitator along with 3–5 participants, each of whom simultaneously connected into the Isness server from one of the nodes shown in Fig. 3. In some cases, additional observers were present within the Isness-D environment: by utilizing ‘spectate’ mode, they were invisible to the participants and to the facilitator. As technical assistants, observers played an important role in helping to stabilize the Isness-D experience. The ‘spectate’ function also helped us to train new facilitators, giving them the opportunity to observe first-hand without affecting the participant experience. All of the Isness-D nodes had available a play space of approximately 2 m × 3 m. To align participants within the shared virtual space, we adopted the following radial orientation strategy: we placed at the center of each node’s space a vector that pointed toward one of the long (3 m) boundaries, aligned each node’s space so that their centers were coincident and the vectors were aligned, and then rotated each node’s vector to make an angle of 360/n° with respect to its neighbors (where n is the number of participants). Figure 3 shows the resulting orientation for our most typical arrangement, with n = 4 participants.

Isness-D was constructed from a set of ‘aesthetic hyperparameters’, each of which controls some aspect of the participants’ phenomenology and can be precisely varied using an available set of on-screen sliders. We defined a phenomenological ‘state’ as a given set of aesthetic hyperparameter values with some specified time duration. Each phase of Isness-D is comprised of a set of states, whose hyperparameter values are saved to a JSON file to ensure reproducibility. The Isness-D experience involved varying 69 different aesthetic hyperparameters, including for example: the color, distribution, density, and latency of the energetic bodies; the size of the heart center light; the rendering options for the energetic thread shown in Fig. 3; options for setting interactive forces to achieve different effects; the state duration, and the global light levels. Our decisions on how to set the aesthetic hyperparameters were grounded in the various design considerations outlined in our previous work^25^. Each phase of Isness-D involved two audio components (played through the HMD earphones): (1) communication with the other participants in the space, which was achieved using an audio-only Zoom call using the mic built-in to the HMD; and (2) a pre-recorded narrative soundtrack, which we streamed as a separate audio channel using Twitch (www.twitch.tv). In what follows we describe the various phases of the Isness-D experience in further detail.

### Pre-experience technical checks

At the beginning of a scheduled Isness-D session, the node hosts would: (1) connect to the cloud mounted Isness server using an I.P. address provided by the session facilitator; (2) connect to an audio-only zoom call, using the earphones and mic in their HMD, and (3) connect to the Twitch feed. After the hosts confirmed that all of the relevant equipment was either plugged in or charged up, they would then enter into the Isness-D virtual environment with the facilitator prior to any participants entering and carry out: (1) an interaction latency check, to identify nodes whose latency or fluidity was significantly different from others as a result of a slow internet connection or network instability; (2) an OMG-VR check, verifying that the gloves generated light through the mudra pose, and were able to interact with the dynamical molecular object; (3) an audio check, where the facilitator ascertained whether any of the host nodes were suffering significant Twitch latency; (4) a spatial orientation check, where each node host confirmed that their respective radial orientation within the Isness-D virtual space minimized cable trip hazards. Hosts were asked to ensure that their participants were not in the same room while these checks were performed; however, this was not possible in many cases owing to space constraints.

We found Isness-D offered a fluid and stable experience so long as the internet connection at each node was stable. Persistent packet loss was the most substantial technological challenge to a smooth Isness-D experience. Technical instabilities were more common during the first week of our studies. Early on, we realized that the fluidity and reliability of an Isness-D experienced depended less on network speed and more on network stability (i.e., reliability low jitter, and low packet loss). We encouraged our nodes to evaluate their network stability using a simple online test^93^, and undertake optimization where possible—e.g., using a wifi router rather than tethering to a phone, moving closer to the wifi router, using an ethernet cable where possible, and encouraging other network users to avoid heavy usage during an Isness-D session. This combination of measures significantly reduced network-related glitches, creating a robust set of distributed nodes, and led to an increase in the number of sessions that we judged to be technologically seamless.

### Phase 1: preparation

Once the facilitator was satisfied that each node passed the technical checks outlined above, they invited each host to fit their participants with the HMDs and a pair of OMG-VRs, and ensure they were in a comfortable seated or kneeling position. When all the participants were ready, the facilitator initiated the Isness-D state sequence and Twitch audio stream. Over the next ~ 15 min (7 aesthetic states), the facilitator: (1) introduced themselves and greeted the participants; (2) asked each of the participants to introduce themselves, saying where they were located physically and how they were feeling; (3) instructed participants to alert their hosts if at any stage they suffered network problems or wished to leave; (4) thanked the participants for participating in our data collection efforts, and (5) asked that everybody respect the anonymity of the others. Next, the facilitator invited each of the participants to stand up and participate in some gentle movement exercises, to help them develop familiarity with moving in Isness-D and interacting with others. Participants were led in the following sequence: (1) acknowledging the others in the space by placing their hands in a prayer position and bowing to one another; (2) three rounds of gently raising and lowering their arms, with synchronized inhalation and exhalation; (3) rapport-building where each participant in turn was offered the opportunity to make up a motion which the others could then simultaneously mimic; and (4) encouraging each participant to gradually move toward the center of the virtual space (“a little closer… a little closer… a little closer…”) until they all overlapped in order to have the experience of coalescing their energetic body with the other energetic bodies (Fig. 3A,B).

The facilitator then invited the participants to return to a comfortable seated or kneeling position, practice generating light using their OMG-VRs, and sculpt the dynamics of the molecular thread. Expanding the scale of the molecular thread, the facilitator encouraged all the participants to hold onto it, creating a circular shape (Fig. 3A.i). The facilitator invited the participants to imagine Isness-D as a kind of meditation on the relationship between matter and energy, and how new senses of interconnectedness might emerge from imagining ourselves, others, and the world around us as luminous energetic essences rather than rigid material objects. The molecular thread was described to participants as a kind of tangible dynamical organism which—because its essence was purely energetic—could connect our energetic essences across different physical locations and time zones. Whilst holding the molecule, the facilitator framed an intention-setting exercise, inviting each participant in turn to say a few words about something to which they would like to connect, inviting them to imagine the molecule as an energetic vessel to contain these intentions.

### Phase 2: A loosely guided choreography

As the visual display of each participant faded to darkness, the facilitator went into ‘spectate’ mode, and another narrator took over. For the next 25 min, participants were guided through a pre-recorded narrative soundtrack played through the HMD headphones. The narrative began with a short visualization, inviting participants to close their eyes and imagine their breath as radiant light concentrated at their heart center. After being instructed to open their eyes, the participants were guided through 16 states, each composed from a different combination of aesthetic hyperparameters. The narrative journey was an adaptation of the experience we previously developed for Isness-C^25^, but reworked in order to accommodate the unique affordances of the distributed environment. The experience took participants through a loose narrative of energetic emergence, dissipation, and fluctuation. It was designed to balance moments of individual introvertive exploration with collective extrovertive interaction. The narrative was accompanied by a soundtrack designed to broadly reflect the arc of the journey. Whilst in ‘spectate’ mode, the facilitator kept watch over the virtual space, in order to discern signs of potential technical difficulties, contact the relevant hosts in cases where there may be technical problems, and gently remind participants who became particularly excited that they should move gently, carefully and fluidly.

### Phase 3: integration & discussion

Once the pre-recorded narrative finished and the participants were once again in a comfortable seated or kneeling position, the facilitator then re-emerged from spectate mode, making themselves visible to the Isness participants and gently greeting them. As during phase 1, the facilitator altered the scale of the energetic molecular organism and encouraged all the participants to hold onto it, forming an energetic circle which connected together the participants, as shown in Fig. 3A.i. Over the next 10 min, the facilitator encouraged the participants to share any thoughts, feedback, emotions, or impressions which arose during the experience. These group discussions were undertaken to enable maximum conversation amongst the participants, with the facilitator’s role as that of an active listener. The format of these group discussions was not rigidly prescribed; instead, the emphasis was on enabling conversation amongst the group. In many groups, participants did not immediately wish to speak. In such cases, the facilitator asked a simple question to initiate the conversation—e.g., ‘How do you feel?’; ‘What remains?’; ‘Were you aware of the others in the space with you?’ Each of these discussions was recorded on Zoom, and subsequently transcribed as part of our data gathering efforts. After the discussion finished, the facilitator instructed participants in how to gently exit VR. Specifically, the participants were instructed to: (1) close their eyes; (2) gently remove their HMDs and place them on the ground next to them; (3) lie down; (4) let the light and sound in the ambient environment seep back into their conscious awareness; (5) open their eyes when they were ready; and (6) undertake the reflective writing on the paper and pencil provided from their host. This anonymized free writing was then photographed by hosts and sent to us for qualitative analysis. Those who were not ready to write in their post-Isness emotional state had the option to do so in their own time.

## Supplementary Information

Supplementary Information.
