# Supplementary material for: Group VR experiences can produce ego attenuation and connectedness comparable to psychedelics
Source: Sci Rep. 2022 May 30;12:8995. doi: 10.1038/s41598-022-12637-z (PMC9149675; doi:10.1038/s41598-022-12637-z)
Supplement: Supplementary file 1 — Supplementary Information. [file 41598_2022_12637_MOESM1_ESM.pdf]

Group VR experiences can produce ego attenuation and connectedness comparable to psychedelics

SUPPLEMENTAL MATERIAL (SM)

This supplementary material includes:

1. **Table SM1**, the mean MEQ30 (**I**, **M**, **P**, **T**) factor scores (as a percentage of the maximum score) for each participant (N = 58).
2. **Table SM2**, *p* values from independent sample t-tests comparing the mean MEQ30 (**I**, **M**, **P**, **T**) factor scores of Isness-D to Isness-C and to previously published YD studies ($\alpha = 0.05$).
3. **Table SM3**, *p* values from independent sample t-tests comparing the mean MEQ30 (**I**, **M**, **P**, **T**) factor scores of Isness-D to Isness-C and to previously published YD studies ($\alpha = 0.01$).
4. **Table SM4**, p values from the 'DistributionFitTest' command in Mathematica, which performs multiple statistical tests to examine whether the data are characteristic of a normal distribution.
5. **Figure SM5**, comparing the average (**I**, **M**, **P**, **T**) factor scores of Isness-D to Isness-C and to previously published YD research studies that have employed the MEQ30, with a 5% significance level ($\alpha = 0.05$).
6. **Figure SM6,** scatter plots of the average ego dissolution against the pre-Isness-D IOS scores for each participant (panel A) and against the post-Isness-D IOS scores for each participant (panel B), with a line of best fit calculated using a linear least-squares regression.
7. **Table SM7,** mean rating and standard deviation for the 8 items in the Communitas questionnaire (items 1–8), and two additional items relating to connection with another participant and with the facilitator (items 9 & 10).
8. **Table SM8,** themes that arose in the qualitative analysis and several corresponding indicative quotations, in order of the number of associated quotations.
9. **Thematic analysis**, the full list of statements from guided group discussions, free writing pieces and post-ceremony questionnaire categorised into each theme.
10. **Isness-D pre-ceremony questionnaire**
11. **Isness-D post-ceremony questionnaire**

Table SM1: Showing the mean MEQ30 (**I**, **M**, **P**, **T**) factor scores (as a percentage of the maximum score) for each participant.

Participant number	Ineffability	Mystical	Positive Mood	Transcendence of Space & Time
**1**	60	53	57	60
**2**	73	67	83	87
**3**	60	77	83	83
**4**	40	45	63	57
**5**	53	65	87	80
**6**	100	61	90	67
**7**	53	35	60	40
**8**	80	35	77	60
**9**	33	55	37	60
**10**	40	53	57	67
**11**	100	51	87	60
**12**	0	45	53	37
**13**	80	55	63	63
**14**	93	79	87	87
**15**	60	63	60	37
**16**	67	48	67	67
**17**	100	97	100	100
**18**	40	52	73	63
**19**	80	75	77	63
**20**	73	75	73	83
**21**	20	27	40	30
**22**	40	37	60	57
**23**	7	17	20	23
**24**	40	27	40	43
**25**	53	44	70	37
**26**	60	60	67	53
**27**	80	71	77	90
**28**	7	13	40	20
**29**	40	48	63	83
**30**	73	32	70	33
**31**	47	11	43	40
**32**	73	64	77	77
**33**	100	83	77	93
**34**	47	31	43	53
**35**	7	21	40	40
**36**	73	60	70	70
**37**	73	48	67	67
**38**	40	56	70	47
**39**	60	60	60	60
**40**	67	41	57	57
**41**	27	71	77	53
**42**	47	13	40	20
**43**	87	87	97	100
**44**	100	83	90	100
**45**	67	52	63	57
**46**	13	3	27	30
**47**	33	8	37	13
**48**	60	60	67	63
**49**	60	53	70	63
**50**	80	49	73	83
**51**	40	25	37	50
**52**	87	48	67	47
**53**	40	29	37	43
**54**	73	43	50	70
**55**	33	27	37	67
**56**	87	63	70	73
**57**	13	5	57	33
**58**	80	87	97	90

Table SM2: The *p* values from independent sample t-tests comparing the mean MEQ30 (**I**, **M**, **P**, **T**) factor scores from Isness-D to Isness-C and to previously published YD studies, calculated by the function "scipy.stats.ttest_ind_from_stats()" (Python 3.7 and SciPy 1.6.2). Studies highlighted in blue were statistically distinguishable for 1 or fewer **I**, **M**, **P** or **T** factor scores and more intense (i.e., higher mean MEQ30 score) than Isness-D. Studies highlighted in light grey are those for which two or more **I**, **M**, **P** or **T** factor scores were statistically indistinguishable from Isness-D. All values that satisfy $p > 0.05$ are in bold and indicate that the corresponding study results were statistically indistinguishable from Isness-D at a significance level of 5%.

		**Ineffability**			**Mystical**			**Positive mood**			**Transcendence of Space & Time**		
	**n**	**mean**	**std dev**	***p* value**	**mean**	**std dev**	***p* value**	**mean**	**std dev**	**p value**	**mean**	**std dev**	***p* value**
**Isness-D**	58	57.2	26.6	n/a	49	22.4	n/a	63.3	18.7	n/a	59.5	21.6	n/a
Isness-C	57	63.9	20.4	**0.13287**	61.5	16.5	0.00092	72.5	11.4	0.00191	66	13.4	**0.05547**
Bar '18, MeO-DMT	20	88.7	12.6	0.00000	79.3	18.4	0.00000	88.7	11.7	0.00000	85.7	13.8	0.00000
Grif '11, psilo (30mg)	18	85	25	0.00019	73	25	0.00024	79	21	0.00344	80	25	0.00113
Grif '18, psilo (20-30 mg) + high M/S practice	25	76.3	19.5	0.00179	71.8	18	0.00002	79.8	19	0.00043	70.6	18.5	0.02796
Grif '11, psilo (20mg)	18	79	25	0.00291	67	21	0.00346	72	25	**0.11682**	69	30	**0.14317**
Nich '18, psilo (42mg)	12	81	26	0.00609	65	35	0.04646	72	28	**0.18513**	73	31	**0.07303**
Carb '18, psilo (30mg)	20	72	18.8	0.02456	61.3	21	0.03471	66.3	20	**0.54511**	59.8	17	**0.95525**
Grif '18, psilo (20-30 mg) + standard M/S practice	25	74.4	31.5	0.01250	60.5	36.5	**0.08258**	74.5	21.5	0.01908	66.6	28	**0.21369**
Nich '18, psilo (31.5mg)	11	78	26	0.01988	60	35	**0.18009**	64	29	**0.91788**	65	31	**0.47436**
Grif '16, psilo (22-30mg)	50	74.5	26	0.00093	59.6	29.8	0.03758	69.8	27	**0.14453**	62.1	23.9	**0.55396**
Nich '18, psilo (21mg)	10	73	27.2	**0.08842**	53	37	**0.64050**	68	28	**0.49964**	54	33	**0.49635**
Carb '18, psilo (20mg)	20	66.3	23.3	**0.17803**	48.5	28	**0.93597**	60.5	24	**0.59372**	51.5	21	**0.15449**
Grif '11, psilo (10mg)	18	66	25	**0.21782**	48	25	**0.87255**	63	25	**0.95651**	47	25	0.04235
Lie '17, LSD (200μg)	11	49	26.5	**0.35166**	44	26.5	**0.51192**	58	26.5	**0.42453**	48	23.2	**0.11415**
Grif '11, psilo (5mg)	18	57	30	**0.97850**	43	21	**0.31728**	55	30	**0.16278**	44	30	0.01823
Lie '17, LSD (200μg)-A	16	83	12	0.00034	40	24	**0.16539**	65	16	**0.74137**	60	20	**0.93391**
Carb '18, psilo (10mg)	20	45.7	19.4	**0.08002**	34.8	19.8	0.01404	49.3	18	0.00468	35.2	16.2	0.00002
Carb '18, dextromethorphan	20	59	18	**0.77973**	29.7	21	0.00117	46.3	22.1	0.00128	49	21.2	**0.06349**
Grif '16, psilo (1-3mg)	50	30.8	31.7	0.00001	24.3	27.1	0.00000	35.8	28.3	0.00000	22.4	20.5	0.00000
Grif '11, Placebo (0mg psilo)	18	23	21	0.00000	19	21	0.00000	33	21	0.00000	21	30	0.00000
Grif '18, psilo (1 mg) + standard M/S practice	25	20.1	20.5	0.00000	13.9	17.5	0.00000	30	18	0.00000	22.3	20.5	0.00000
Carb '18, Placebo	20	4.7	9	0.00000	6.5	9.3	0.00000	15.8	11.7	0.00000	6.3	9.8	0.00000
Lie '17, MDMA (75mg)	30	16	16.4	0.00000	5	32.8	0.00000	18	21.9	0.00000	9	27.3	0.00000
Lie '17, Placebo (25μg LSD)	4	3	0	0.00015	4	0	0.00018	9	8	0.00000	5	2	0.00001
Lie '17, methylphenidate (40mg)	30	7	16.4	0.00000	1	0	0.00000	11	21.9	0.00000	4	0	0.00000
Lie '17, Placebo	16	0	0	0.00000	1	0	0.00000	3	0	0.00000	2	0	0.00000
Vlis '18, ketamine	15	6.5	2.6	0.00000	-	-	-	-	-	-	6.6	2.1	0.00000

Table SM3: Identical values as to those **Table SM2**, except the formatting indicates statistically indistinguishable studies using a significance level of 1% (*p* > 0.01).

		**Ineffability**			**Mystical**			**Positive mood**			**Transcendence of Space & Time**		
	**n**	**mean**	**std dev**	***p* value**	**mean**	**std dev**	***p* value**	**mean**	**std dev**	**p value**	**mean**	**std dev**	***p* value**
**Isness-D**	58	57.2	26.6	n/a	49	22.4	n/a	63.3	18.7	n/a	59.5	21.6	n/a
Isness-C	57	63.9	20.4	**0.13287**	61.5	16.5	0.00092	72.5	11.4	0.00191	66	13.4	**0.05547**
Bar '18, MeO-DMT	20	88.7	12.6	0.00000	79.3	18.4	0.00000	88.7	11.7	0.00000	85.7	13.8	0.00000
Grif '11, psilo (30mg)	18	85	25	0.00019	73	25	0.00024	79	21	0.00344	80	25	0.00113
Grif '18, psilo (20-30 mg) + high M/S practice	25	76.3	19.5	0.00179	71.8	18	0.00002	79.8	19	0.00043	70.6	18.5	**0.02796**
Grif '11, psilo (20mg)	18	79	25	0.00291	67	21	0.00346	72	25	**0.11682**	69	30	**0.14317**
Nich '18, psilo (42mg)	12	81	26	0.00609	65	35	**0.04646**	72	28	**0.18513**	73	31	**0.07303**
Carb '18, psilo (30mg)	20	72	18.8	**0.02456**	61.3	21	**0.03471**	66.3	20	**0.54511**	59.8	17	**0.95525**
Grif '18, psilo (20-30 mg) + standard M/S practice	25	74.4	31.5	**0.01250**	60.5	36.5	**0.08258**	74.5	21.5	**0.01908**	66.6	28	**0.21369**
Nich '18, psilo (31.5mg)	11	78	26	**0.01988**	60	35	**0.18009**	64	29	**0.91788**	65	31	**0.47436**
Grif '16, psilo (22-30mg)	50	74.5	26	0.00093	59.6	29.8	**0.03758**	69.8	27	**0.14453**	62.1	23.9	**0.55396**
Nich '18, psilo (21mg)	10	73	27.2	**0.08842**	53	37	**0.64050**	68	28	**0.49964**	54	33	**0.49635**
Carb '18, psilo (20mg)	20	66.3	23.3	**0.17803**	48.5	28	**0.93597**	60.5	24	**0.59372**	51.5	21	**0.15449**
Grif '11, psilo (10mg)	18	66	25	**0.21782**	48	25	**0.87255**	63	25	**0.95651**	47	25	**0.04235**
Lie '17, LSD (200µg)	11	49	26.5	**0.35166**	44	26.5	**0.51192**	58	26.5	**0.42453**	48	23.2	**0.11415**
Grif '11, psilo (5mg)	18	57	30	**0.97850**	43	21	**0.31728**	55	30	**0.16278**	44	30	**0.01823**
Lie '17, LSD (200µg)-A	16	83	12	0.00034	40	24	**0.16539**	65	16	**0.74137**	60	20	**0.93391**
Carb '18, psilo (10mg)	20	45.7	19.4	**0.08002**	34.8	19.8	**0.01404**	49.3	18	0.00468	35.2	16.2	0.00002
Carb '18, dextromethorphan	20	59	18	**0.77973**	29.7	21	0.00117	46.3	22.1	0.00128	49	21.2	**0.06349**
Grif '16, psilo (1-3mg)	50	30.8	31.7	0.00001	24.3	27.1	0.00000	35.8	28.3	0.00000	22.4	20.5	0.00000
Grif '11, Placebo (0mg psilo)	18	23	21	0.00000	19	21	0.00000	33	21	0.00000	21	30	0.00000
Grif '18, psilo (1 mg) + standard M/S practice	25	20.1	20.5	0.00000	13.9	17.5	0.00000	30	18	0.00000	22.3	20.5	0.00000
Carb '18, Placebo	20	4.7	9	0.00000	6.5	9.3	0.00000	15.8	11.7	0.00000	6.3	9.8	0.00000
Lie '17, MDMA (75mg)	30	16	16.4	0.00000	5	32.8	0.00000	18	21.9	0.00000	9	27.3	0.00000
Lie '17, Placebo (25µg LSD)	4	3	0	0.00015	4	0	0.00018	9	8	0.00000	5	2	0.00001
Lie '17, methylphenidate (40mg)	30	7	16.4	0.00000	1	0	0.00000	11	21.9	0.00000	4	0	0.00000
Lie '17, Placebo	16	0	0	0.00000	1	0	0.00000	3	0	0.00000	2	0	0.00000
Vlis '18, ketamine	15	6.5	2.6	0.00000	-	-	-	-	-	-	6.6	2.1	0.00000

Table SM4: The p values returned from the 'DistributionFitTest' command in Mathematica on the **I**, **M**, **P** and **T** averages for each participant, where the p value indicates whether the data are characteristic of a normal distribution. The data were categorized as being normally distribution if the p value was above a statistical significance level of 5% ($p > 0.05$). The **M**, **P** and **T** factor scores pass every test, and the **I** factor score passed all except the Pearson χ^2 test (in bold).

	**Ineffability**	**Mystical**	**Positive Emotions**	**Transcendence of Space & Time**
Anderson-Darling	0.12434	0.61534	0.07552	0.53278
Baringhaus-Henze	0.45081	0.56455	0.20420	0.90626
Cramér-von Mises	0.16477	0.55253	0.09207	0.49145
Jarque-Bera ALM	0.31246	0.58568	0.48709	0.59613
Mardia Combined	0.31246	0.58568	0.48709	0.59613
Mardia Kurtosis	0.30232	0.38436	0.35291	0.33066
Mardia Skewness	0.38654	0.63793	0.54530	0.90848
Pearson χ^2	**0.03311**	0.80075	0.12823	0.39732
Shapiro-Wilk	0.07108	0.57495	0.17330	0.45009

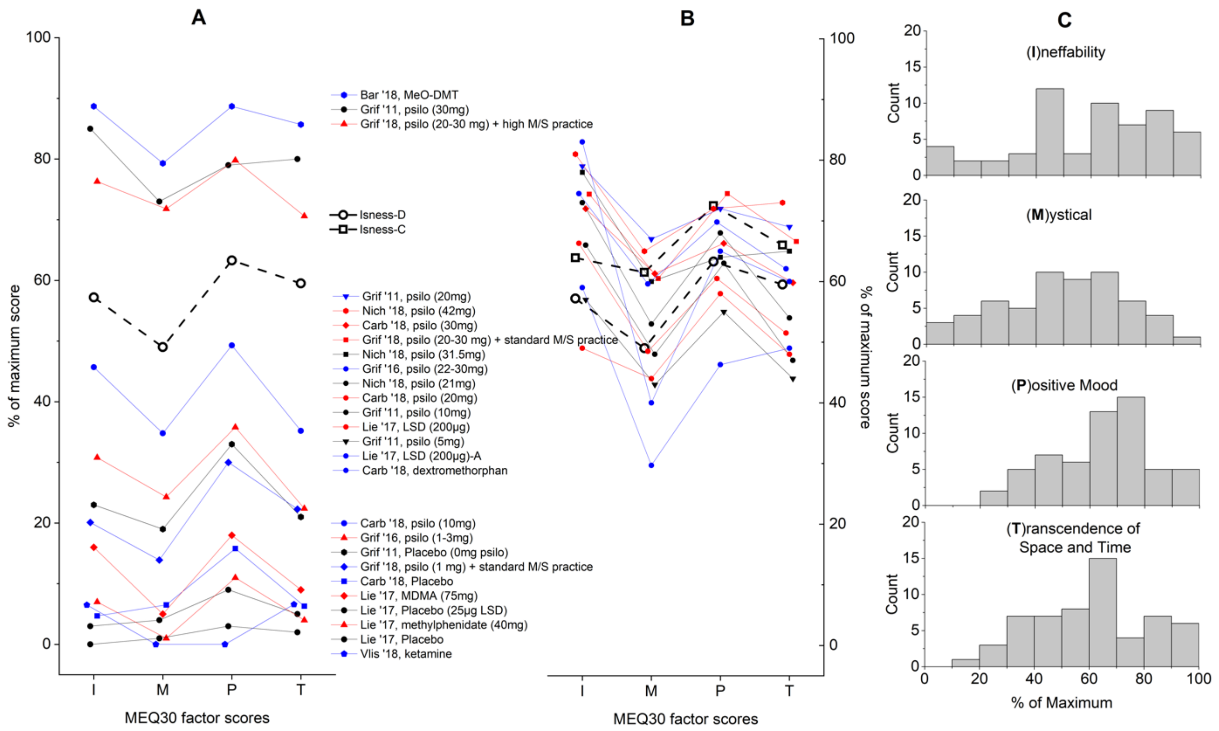

Figure SM5: Comparison of the average (**I**, **M**, **P**, **T**) factor scores of Isness-D to Isness-C and previously published YD research studies that have employed the MEQ30. Panel (A) shows the studies that were statistically distinguishable from Isness-D ($p < 0.05$); (B) shows studies which are statistically indistinguishable from Isness-D for 2 or more of the (**I**, **M**, **P**, **T**) factor scores; and (C) and C shows the distribution of the four factor scores of the MEQ30 results of Isness-D.

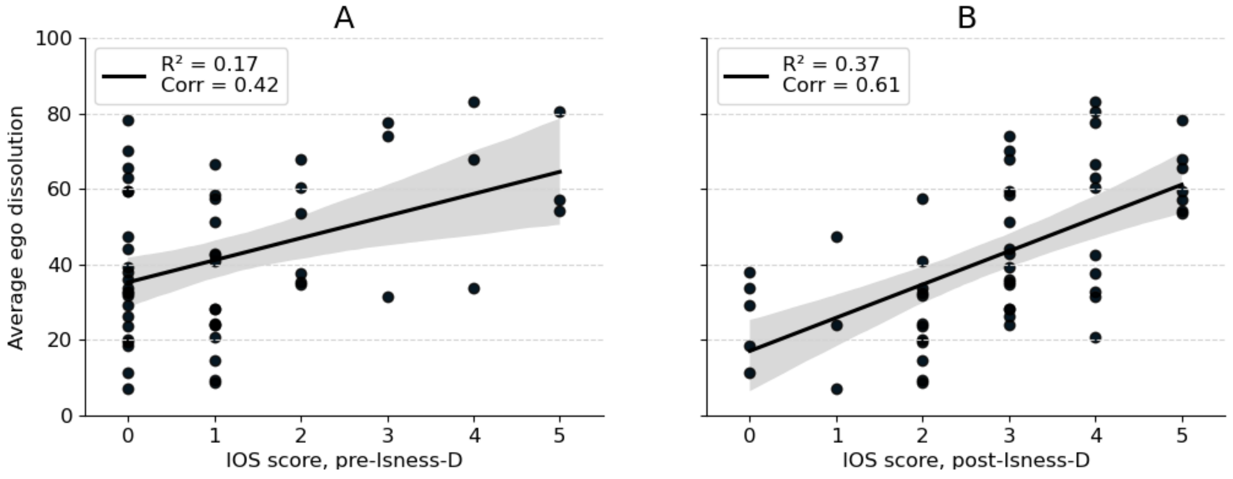

Figure SM6: Scatter plots of the average ego dissolution against the pre-Isness-D IOS scores for each participant (panel A) and the post-Isness-D IOS scores for each participant (panel B), with a line of best fit calculated using a linear least-squares regression. Each legend gives the correlation coefficient and the R^2 value.

Item number	Item	Average	Std. dev.
**1**	During the ceremony, I felt a bond with my fellow participants that I could not experience outside the ceremony.	5.2	1.6
**2**	During the ceremony, I felt a sense of belonging with the other participants.	5.5	1.2
**3**	During the ceremony, I felt a sense of harmony with the others.	5.9	1.1
**4**	During the ceremony, I felt a sense of sharing with the other participants.	6.0	1.1
**5**	The ceremony really allowed me to get to know the other participants.	3.6	1.4
**6**	During the ceremony, I felt that social status became irrelevant.	6.4	1.0
**7**	During the ceremony, I experienced all participants as equal based on basic shared humanity.	6.2	1.3
**8**	During the ceremony, ego-related tensions between participants seemed to resolve.	5.3	1.4
**9**	During the ceremony, I felt a strong connection to another participant.	4.3	1.9
**10**	During the ceremony, I felt a strong connection the facilitator.	4.6	1.5

Table SM7: Mean rating and standard deviation for the 8 items in the Communitas questionnaire (items 1–8), and two additional items relating to connection with another participant and with the facilitator (items 9 & 10).

Table SM8: Themes that arose in the qualitative analysis and several corresponding indicative quotations, in order of the number of associated quotations.

**Theme (N statements)**	**Indicative quotations**
**Connectedness (75)**	- I'm pretty bewildered… It's very immersive… you lose yourself in the connection to other people.<br>- It was the antithesis of what we're going through right now, with all the separation.<br>- *Blissful sense of connectivity.*<br>- You can have this quite intense… intimacy in space… but you are also totally safe. You can be with strangers and… be really close but also know where your boundaries are.
**Positive emotions (65)**	- I feel remarkably happy. There's something very happiness-inducing about this whole practice.<br>- It was quite a trip. But a pleasant one.<br>- For me that was very very calming, amazingly calming.<br>- I feel way less stressed.
**Embodied awareness (40)**	- Even though there was no body there, you wanted to respect each other's space.<br>- It felt really nice to touch people. Having not touched people for so long [in lockdown], it felt really nice.<br>- *Following the [dynamical molecular organism] with my nose, it felt like I was breathing it in.*
**Ego dissolution (31)**	- Identity didn't matter anymore; it was about experiencing things together. That was wonderful.<br>- I found [the anonymity] quite powerful because we were all completely equal in the space. Any of the pre-judgements that come in from how people look, sound and that sort of thing just aren't there.<br>- Yeah, I felt [when touching one another's heart centres] the sweetest tenderness or pure, childlike love… stripped back, without any of the assumed layerings that we place upon reality and relationships… just to the absolute core, it was truly beautiful. Thank you, all of you. Thank you, Isness.
**Supportive setting (28)**	- At first, I thought [it] might be difficult to [reflect on connection with] strangers, but there was no pressure to give a long statement… the breathing exercise helped get comfortable in the space.<br>- *I really liked the opening and closing exercises with [the facilitator]. It really helped to hold the experience and make me feel more comfortable with everyone.*<br>- *The time of reflection at the end of the session, hearing other people's thoughts on the experience made the experience more meaningful.*
**Sense of play (24)**	- There certainly was a sense of playfulness in this, which was really nice. As adults that's not always something that we pursue. It was good.<br>- I was inspired to dance on the top of the table at one point.<br>- *A feeling of being grounded, a playfulness facilitated in a calm way.*
**Transcendence of space/time (13)**	- It felt like the space opened up to me. I'd been aware of the confines of [the space], vaguely. And then we pushed [the molecular organism] through the floor… it was very odd to feel like everything opened out.<br>- Things didn't have the same human scale, so at times it felt this could be [an enormous] space and we were a completely different scale, or it could be microscopic, and it didn't really matter, actually.<br>- *I liked the parts that allowed me to view the world in a way that isn't possible in real life.*
**Noetic quality (11)**	- I got quite emotional… And then the experience was guiding us forward, so it was fleeting. But I got this surge of emotion where I don't know if I wanted to gasp or cry or what it was, but I was kind of shocked in awe.<br>- That's definitely a spiritual experience of some sort. It's, like, tangible.<br>- *Pure unity.*
**Comparison to other altered states (10)**	- [Taking psychedelics] wasn't quite like this, but I guess there's elements of it, for sure.<br>- *I experienced the sense of interconnectedness…only previously noticed with the help of psychedelics in the right setting.*<br>- *The experience brought up memories for me of medicine plant journeys and realisations that have shaped my life and give my life meaning. These realisations are very much connected with going beyond form...*
**Sense of beauty (9)**	- I was amazed [by] how moved I was. I think it was the juxtaposition of the frustration and then the beholding beauty. I think the music really helped. It felt very beautiful to sit back and witness. I don't normally get moved quite so much.<br>- Yes, thank you. I was a little bit doubtful in the beginning, I didn't know what to expect and what was going to happen. It was a beautiful experience.
**Reflection on mortality (8)**	- It's clear that [our life] energy isn't just gone, it goes somewhere. It's beautiful to think that it's all out there somewhere and it's still circling you constantly<br>- There was one moment… talking about seeing past energies and I was thinking about my cat, whom I lost last year. I really miss her. I could really see her and… was writing her name. It was beautiful, thank you.

THEMATIC ANALYSIS

A summary of the identified themes (N quotes):

1. Connectedness (75)
   a. General (42)
   b. Through body-movement (19)
   c. Intimacy (14)
2. Positive emotions (65)
   a. General (40)
   b. Feeling of calmness (25)
3. Embodied awareness (40)
4. Ego dissolution (31)
5. Supportive setting (28)
6. Sense of play (24)
7. Transcendence of space and time (13)
8. Noetic quality (11)
9. Comparison to other altered states (10)
10. Sense of beauty (9)
11. Reflection on mortality (8)

Each quotation has been formatted according to the source:

- Regular = Guided discussions
- **Bold = free writing responses**
- *Italic = questionnaire feedback from "what did you like?"*
- ***Italic bold - questionnaire feedback from "Do you have any other comments?"***

Theme: Connectedness

General connection

1. At first I was a bit suspicious with this network, but I feel that I'm starting to understand the metaphor a bit better...The one thing that I was a bit wary of was the fact that we had to go about and do the mudra in order to interact with it. I thought at first it would go against the idea of being always connected, but I guess now I feel like in order to be connected we have to put in effort, we have to understand that. Being connected is not something that should be expected all the time. And it takes some effort to realise it, but now I feel like, I don't know if that's going to be a changing experience, but now I'm ready to have this metaphor in my head whenever I'm scouring through life.
2. Yeah, when we were doing the initial mudra and [the narrator] said change to the other one and see what you notice. But that initial one, yeah, I could just get a real connection with [the molecule].
3. I feel very calm and centred...It's really nice. Connected as well, you know.
4. I've been also trying to be more connected with myself through meditation, and it felt really nice to have a meditation with other people. So, I felt connected with myself but also with everyone else here. It's an experience I haven't felt in a while because of COVID and being in front of screens all the time. That's why I think 'connected' is the word for me for the end of this session.
5. This is the closest [that] I've been to any strangers in a year.
6. It was beautiful. It was amazing. I kind of feel like I know you guys now.
7. It felt more useful [than other guided meditation], in a sense a bit more connected.
8. I think it's nice that we're all in the middle together now. We all started out on the outside and we're all together now, it's nice.
9. I suppose it's a bit more collaborative than just a normal meditation because we were able to work together and connect with each other in different ways.
10. I'm pretty bewildered, to be honest. It's very immersive. It feels you lose yourself in the connection to other people. Quite remarkable.
11. Yeah, I feel grateful as well, for being so connected to the sense beyond touch and taste and smell. Very clear connection in a way [trails off] beautifully connected in this experience.
12. It was also just so amazing to feel that movement in myself to connect, and that readiness. All I wanted to do was to be together. Such enjoyment of being together and to feel that so powerfully in oneself, that is really confirming and opening and expanding.
13. Such a pure joy of connection.
14. Connection for sure, yes, and so much more.
15. [When the lights faded] Still connected.
16. Especially in the situation we're in at the moment, globally, not only was there, on a practical level, more freedom to move and [no] fear that you were going to whack someone in the face. But also, you could physically get closer to one another's lights, which was really cool. You could be in the light, which you couldn't before. But also, the connection is so much more than who you're physically in the same room as, it's about a bigger thing, feeling connected to people we're not physically connected to, necessarily.
17. I wish it was bigger, I don't know if that makes sense. That there was more space and, also, more of us...It felt like at any moment the lights could come on and there would be 6 billion more.

18. I think the fact that we're all doing it together makes such a difference...It's the fact that you realise that it is someone else, an actual real person making those moves.
19. It was the antithesis of what we're going through right now, with all the separation.
20. It was weirdly connected [when coalescing].
21. **Connecting light and emotions as energy was special. My work is in renewable energy and I also take photographs and think about light; light as connection; light transferring between matter; light creating memory. Experiencing myself and the other people in the group as light energy was joyful. It was both another space and allowed me to think about other spaces connecting in the world.**
22. **Loved the thought of connection with total strangers, in such a gentle way. Thank you for the experience!**
23. **It was really nice to feel really connected to people – especially during COVID to feel so free to touch & connect with other people. Felt very strange standing inside someone, but also lovely.**
24. **I noticed that I enjoyed the ability to join with people and leave when I wanted — togetherness and separateness. The option does not always seem available to choose for myself. The music was a significant part of the experience for me — relaxing and connecting. The attention to a separate 'self' was intriguing. The energy part. The energy among the lights, (people) was easier for me to experience — I experienced some type of connection.**
25. **I liked the fogging effects that connected us all in haze. Very soothing and centering experience overall. I was sort of expecting it to go from protein folding to a tree growing. I liked us being amorphous jelly fish together. Feeling connected to others —distance irrelevant.**
26. **Actually, this great 'therapy' in time of social distancing. Happy to participate again and again.**
27. **Connectiveness — good moment of release.**
28. **Wished to see and feel all the people on Earth in the experience, since the idea is that we are all light beings. Would be incredible to experience that directly.**
29. **I felt at ease with the other participants and felt a positive connection with them at the end.**
30. *Blissful sense of connectivity.*
31. *Everything! I liked being energetically Together yet Physically apart from the other participants a lot. It increased the sense of connection.*
32. *I felt excited by the international connections and the use of the Internet as a way of creating relationships between people whom I have never met. I felt a sense of joy and connection which, in part due to lock down, I have been unable to experience as my normal dance practice of contact improvisation has been suspended.*
33. *That I was able to feel immersed in the experience. Feeling of connection with a group of strangers who were spread out across the country.*
34. *The potential for bringing people together.*
35. *Connecting with the other participants, interacting with beings as balls of light.*
36. *The opportunity to connect with other beings and myself in a new way.*
37. *The feeling of calm and peace I felt and the thought that I was connecting with strangers from around the World going through the same experience.*
38. *I like that I have a visual now that I can map onto the real world when I'm around other people so that I can see everyone as that beam of light.*
39. ***I wish I could do it again, now I know what's involved. It was so beautiful; I felt an amazing connection to the lights and energy. Now I think about it, maybe I did feel more 'at one' than I scored in the questionnaire.***

40. ***Really great, I would love to do this with my siblings across the world as I feel it would make me feel so close.***
41. ***Really beautiful. My intention at the start was to look more at my connection with myself and my body and I really feel like I was able to explore this from a new and different point of view. Thanks guys!***
42. ***I think I preferred the remote experience more than when we were in the same room. It felt more profound and in line with the project. I also wasn't afraid I was going to whack someone and so I could move more freely. It also meant we could get closer than before which felt more intimate, and connecting—nearly as much so as with a partner, child or pet—even though we were in different places.***

Connection through body movement

1. I think I was still aware that I wanted to help out what everyone else was doing, rather than ruining it, if that was such a thing. If someone was trying to go a certain direction, then I wanted to go with them rather than spoil their activity.
2. Today felt so much more like a dance floor. I guess you feel that thing when you're on a dance floor in a club with your mates and you feel that sense of connection. And you're moving in a similar, exploratory way. Today, for some reason, [Isness] felt more like a disco.
3. I just wanted to move in, to be honest. I was just really curious to see what happens when you put more and more light together. And I think people were receptive, I don't know if that's just in my head. When I was trying to get as much light together as possible, I felt like a lot of people were following those same thought patterns. And you definitely felt that when we were all bunched up on top of each other, that was really cool.
4. I felt really connected with all the other people in this room, even though we're in different places. It was really nice all putting our lights in the middle, it was a way of feeling connected.
5. It felt like there was a way to tell whether people wanted to [coalesce] or not, people kind of moved gently and moved direction.
6. Naturally, I wanted to start moving slower. And I felt like the interaction could be greater perhaps when we were moving slower. So, I feel like I slowed down during the process. I started a bit more like 'woah, what's going on', and then I was enjoying slowing down and observing others and seeing how others were interacting if I went near them.
7. I also found it really cool as a social experiment, to see how leading and following happens within the group before it started being led. When we were just left to our own devices, what we implicitly did with the creature—trying to stretch it out—came from someone and everybody else just picked it up. How quickly that happened was fascinating.
8. **Really enjoyed that. Loved feeling of being connected with people — sense of a very intimate space despite being so far apart (one in London and one in Knowle, Bristol). Lovely working together. You get a sense of whether others in the group are playful, generous, co-operative, from how they interact with the shapes and the other people. So — body language still comes through even without a physical body.**
9. **OK, trying not to think too much but I can't help it. I'm really into the idea of building the sense of interconnectedness in this way. Simple; three moving lights to represent a stranger. Was enough to really feel and play with a sense of connection. There was mirroring, suggesting/influencing, and being influenced. Plus a shared novel experience.**

10. **Social experiment – play and unspoken coordination. I really liked how quickly unspoken coordination to stretch out the creature developed – way before we got 'the directive'. The directive then felt really out of touch.**
11. **I didn't really connect with the 'beings of light' script. Instead, I thought the participants convinced me they are good, well-intentioned, and bright people ('that carry light') all on their own, through interaction. The whole recorded script fell flat in the light of this realization that was about reality, not some pre-recorded open-loop non-judgement.**
12. **Doing simple actions, having to go into the middle, choosing a movement helped put me at ease. Guide stating to be gentle with the molecule set up the conditions of we should interact in the space – 'be gentle'. And each other. There was a sense of play which you don't have in the real world because of social constraints. I felt everyone was respectful of each other's space, no one was dominant, more curious and wanting to gently interact.**
13. *I tend to be quite shy but felt I got easily and quickly comfortable with the others, through body movements and interactions mainly.*
14. *I loved playing with the energy and manipulating it. It was great when we could all coordinate our movements.*
15. *I enjoyed playing particularly with one of the participants, improvising and guessing each other's moves.*
16. *That there were a small number of participants, so we could interact in a small group. Everyone was friendly and playful. The visuals, especially the trails and fog effects were cool. Working together to stretch the protein/organism/thing out was fun.*
17. *The way it became a dance.*
18. *I felt free to dance with others and play freely.*
19. ***I feel much closer to my intention set at the start than I did before the session. I enjoyed the live guiding and group engagement a lot and felt very safe and connected. The pre-recorded guide sometimes seemed too frequent or didactic so there wasn't as much space as I would have liked to play, explore, learn from watching others through chance encounters.***

Connection in intimacy

1. It feels really much more intimate, so much more intimate [than a video conference].
2. The fact that you can have this quite intense, almost, intimacy in space, you are right in each other's space, but you are also totally safe. You can be with strangers and you can be really close but also know where your boundaries are in certain ways.
3. It felt really intimate. And I tried to connect whichever light being it was, and you didn't know who it was. I tried to join mudras, and I think I sat inside a few of you. I hope that was OK!
4. It's also really interesting because the first time I tried this there were four people in the room together, so we ended up bumping into each other and you had to be aware of space and cables. And now, I can just literally walk into people and it's quite sensuous, almost.
5. It felt strangely intimate...it was really nice.
6. **Feel slowed down now. Liked connecting with others. More intimate than would have thought. Enjoyed the visuals of my own movements, was less interested in others.**
7. **Overlapping in 'virtual space' almost felt uncomfortably intimate at time—probably a good exercise!**

8. **Physical boundaries being breached was initially really jarring but quickly gave way to feeling of intimacy. This was a really good part of the experience.**
9. **Super fast intimacy and trust establishment. Following and leading, playing one on one led to a feeling of true intimacy, which was a surprise. Was completely emergent. I used to do improv – this kind of exercise would establish physical trust much faster than happened in real life.**
10. **I found it incredibly energizing. Crawling through the light, the creature we were all holding, as well as the [?] light of other beings. It was sensual and playful and safe, felt like feeding off each other's energy. Also, quite erotic.**
11. *The sense of an intimate space despite being far apart.*
12. *The closeness, intimacy, body-meld.*
13. *The sense of intimacy.*
14. ***I thought the playful intimacy did come about very quickly, I have experienced real life playful intimacy through Contact Dance to the same kind of depth however, I would agree and echo one of the other participants that this happened very quickly.***

Theme: Positive Emotions

General

1. This was amazing.
2. And just I guess a feeling right now, not that it was part of the experience, but just a great conversation with a bunch of glowing gas balls. Absolutely loving this, it's something I never knew I needed.
3. Just a whole lot of gratitude, [Facilitator's name]. This has been absolutely gorgeous.
4. Yes, thank you so much for this opportunity, I never felt like this before. And thank you for that.
5. When [the molecule] was leaving behind trails, I found that particularly amazing. That really felt extraordinary.
6. Yeah, [coalescing] feels a bit funny at first, quite jaunting almost. But then it feels quite nice, like 'ah yeah, OK!'
7. I feel like there's a difference between stagnant and stillness. That's how I'm feeling. That it's nice to know the difference. I guess this time can make you feel very stagnant, cause you're in the same place and you're not still or rooted to anything...I feel like there's a difference.
8. I liked [coalescing], I thought that was really nice.
9. [Coalescing] was great.
10. [Coalescing was] a quick way of freeing you up and making you realise that you can walk across other people's space.
11. I was expecting to walk into you guys, but [we were] disincorporated beings. It was great.
12. [Coalescing] was a highlight, an absolute highlight. It was very interesting having done the physical version of this and seeing the advantages of the virtual. That was definitely one of them, being able to invade and conquer someone else's space.
13. I like silence in the space. It's really interesting the experience of silence in this dark space without anything at all. Not only the experience of light, but [also] the experience of silence and darkness is really great.
14. Yeah, I was going to say that I haven't really got any words. Which is a good thing, I think.
15. [Coalescing] was definitely a highlight.
16. [Coalescing] was really cool.
17. I felt quite greedy for it [the molecule]. I just wanted to be in it and it to be completely surrounding me and to be amongst it. I still feel that.
18. I feel remarkably happy. There's something very happiness-inducing about this whole practice.
19. I feel happy and pretty optimistic about going out and seeing energy in everything and having that as a positive lens about life.
20. It was quite a trip. But a pleasant one.
21. **I'd like to do again in the morning, though curious what I'll be in dream state tonight.**
22. **I really like that the creature is common to all.**
23. **Felt a strong urge to disappear/let go of mudras. When I did, I felt elated and giggly.**
24. **Overall, very impressed. Thanks to everyone who worked on this, it's going to be a beautiful, revolutionary public service.**
25. **The previous time I experienced this, there were people actually in the room with me and I was conscious of their personal space, headset cables, etc. Now it was so much more freeing and … safe?**
26. **Initially I was thinking about driving on the motorway with other death machines around me, which makes me anxious. Translating that sense of danger in a completely safe space, yet with other live participants who you can still FEEL almost physically, I was delighted that it didn't diminish the sense of sharing energies at all. That is amazing!**
27. **I feel so [?], awake, glowing, even after a long day. Transcending time, time zones, other humans in the experience with me … I feel so full of LIGHT and HAPPY.**
28. *All of it.*
29. *All of it!*
30. ***It was a really GORGEOUS experience... this iteration is more powerful than the Church one I did 8-9 months ago…the music adds a lot…***
31. ***Great experience, thank you.***

32. ***It was very centering!***
33. ***Brilliant.***
34. ***I really enjoyed it, and it is something that I would definitely like to do again in the future.***
35. ***No. It was fantastic! :)***
36. ***Thank you. I'm humbled and grateful to have been able to enter this space. It arose so many things from within and beside me. Thank you! Very fun and very humbling.***
37. ***I had a great time, but mostly forgot my 'connectedness intention' that I had been asked to think about before the isness experience as I played with the energy string and the other participants. I was aware from conversations with others about the inspiration from chemical modelling so that may have been in my mind when I did that.***
    It is something I think I will remember for a long time, and would love to have another play!
38. ***I never thought this could be done with VR. Amazing. Please keep going.***
39. ***Many thanks! Amazing :)***
40. ***I really enjoyed it!***

Feeling of Calmness

41. Tranquil.
42. Yes, absolutely [I feel chilled]. I'm like a lukewarm glass of water.
43. [When the lights faded] Like going to sleep as a kid. Maybe at the end of your birthday, or something. Or falling asleep in the car when your parents are driving.
44. [When the lights faded] Like when you're little and you fall asleep and your parents are having a party. You know there's those times where you fall asleep on the sofa and could hear other voices around you and that sense of togetherness.
45. And I definitely want to do it again. Now I've experienced it, I want to do it all over again and I can relax into it a bit more. I do feel relaxed now. Especially that point where our lights all got on top of each other. It was relaxing.
46. I feel way less stressed.
47. Yes, very relaxed. Really calm. And I was amazed at how I wanted to gather all the mudras together. I wanted to bring everyone [trails off]. I didn't like to see people straying off.
48. I felt very peaceful, I think.
49. For me that was very very calming, amazingly calming.
50. I will say that I'm relaxed in a way that I can describe, I think that my shoulders are very much lower, I had a tense neck. And I loved the dance, I was dancing with my hands.
51. **Overwhelming calmness once I had let myself fully immerse in the journey!**
52. **The experience was fun and eye opening! Felt very meditative and calming. It was cool interacting with the 'organism' as well as the fellow users.**
53. **Overall, a very joy-inducing and calming experience.**
54. **My first VR with headset. Reminiscent of Tokyo Light Digital Museum. Very relaxing (recharging).**
55. **All in all, I felt renewed/re-energized. [?] Would be happy to do again.**
56. **Heart opening.**
57. **Great for group therapy. Great for group tonglen practice.**
58. **There was a point when you were asked to focus on the past as you looked at the light trails. I had a spike of an anxious feeling – perhaps because depression is an obsession with the past…and I have always looked at it in a way where I focus on the negative. What was interesting was I didn't feel like that; I focused on the non-linear patterns. It was nice to look at the past in a calming way.**
59. **I found it difficult to write about this experience straight afterwards. Although I felt lucid and wanted to talk to my host about it, I have had very limited experience with VR and was focused on feeling calmer and strangely energized from the experience.**
60. **Some of the questions in the survey were difficult to answer because I didn't come into the experience with an ego – my starting place was a feeling of anxiety and stagnation. I came away from the**

experience with an overwhelming sense of calm, which onto the next morning. I woke up thinking about how I keep a sense of play and wonderment.

61. *Interacting with the energy string was fascinating and soothing.*

62. *The use of VR to aid the disillusionment of self as a starting part to a meditative practise.*

63. *I liked the immersive nature which removed me from my normal thoughts and experiences.*

64. *Calming, relaxing feeling at end.*

65. ***I'm not sure I took away any profound insights, but this may be because I have never really tried yoga or meditation, which would perhaps make me more primed and open for this type of experience. It was, however, quite relaxing and I would try it again.***

Theme: Embodied awareness

1. I almost…we were different creatures. In fact, I remember now the dance movements were these fuzzy, round...you know...we are these fuzzy round cloudy things, and that was a great form for the particular dance.
2. It felt like we were all, you know, coming together, like I could *feel* that. That's what it felt like, I think.
3. Having done mediation without visual aids, I've not really done group meditation before. It's a similar process although I definitely feel an awareness of other people in my space. There's a duality in the fact that you're not in my physical space. Definitely, I think that at the end when I closed my eyes, I could still feel things moving around me. It's hard to separate traces that are left on my visual senses, differentiation the physical from the mental.
4. I thought it was very interesting to experience the same space without bumping into anybody. And just becoming into the same space with other people and merging, sort of, and at first I feel a bit 'OK I'm going to bump into them' but no, I can completely merge with them, it's wonderful.
5. Even now, I'm arranging myself sat down and I'm like 'ooh have I given space to the person on the left and the person on the right' and it's kind of incredible.
6. There's definitely a feeling here like 'someone's messing with my lights' [and] 'somebody's standing in my orb' or 'somebody's standing in my space' or 'am I going to touch someone else?'. Like 'is that allowed?' [or] 'is that taboo?'.
7. It felt really nice to touch people. Especially in lockdown, having not touched people for so long, it felt really nice.
8. I think I still found myself respecting peoples' personal space; it felt weird to try and walk over someone or walk through someone. I did treat people as if they had a slight solidness to them.
9. I found myself putting my ball of light on other peoples' hands and then feeling self-conscious that I was an inappropriate guy at the party.
10. I totally stepped into all of you purposely, I wanted to see how that felt like. I was trying to put my head on your other heads and my hands in the same spots, just to see if that made me feel anything. It felt like someone else's energy.
11. I always find it interesting when you give people more space to these, let's say, 'imagined people' than you would in real life. You can sense an imaginary presence around the glowing light, and you give it space even though there is no barrier there, other than imagined. But, with people in real life, even though it is, let's say, 'real' or the barrier is real, you push that more so than you would here, where you give people more space.
12. Even though there was no body there, you wanted to respect each other's space.
13. I wasn't sure if something else was going to happen when we did that [coalescing]. I don't know what I was expecting. It was weird, I suppose, not touching because it feels like we should have been, if you see what I mean.
14. I think the 'not touching' is part of starting the dissolution of the body and translation into this kind-of more 'energy state', and so it felt, when you couldn't touch someone, you were like 'that makes sense' because you're no longer a body, but you're light.
15. I found it really strange at the start when you were telling us to go closer and closer and then that weird boundary of where personal space is...and all of a sudden it was like 'oh, actually this is kind of nice!'. Like, we're all in the same block together.
16. Yeah, [coalescing] was really nice.

17. I felt aware of that, I think, more so than I needed to, being in someone's space.
18. I think part of me, I don't know, there was something in me that felt a little bit rude walking into someone else's space, despite the fact that I'm not actually doing that. It felt quite intimate with strangers.
19. I kept wanting to touch people, physically. To reach out and grab their hands or hug them, see what would happen if our energy came together.
20. I actually had a sensation of different temperature on my skin. And my heart opened. It was definitely heart-opening. Much more rapid, I think, from the regular loving, kindness meditation, even. It speeded up the heart-opening for me a little bit. Yes, I felt really deeply connected to all of you. In a human but non-human way.
21. When we could then, right at the start, we all moved closer and closer together and we [were] on top of each other's lights. Until that point it really felt like you were all in the room with me, in a sense, and then that made me realise that you weren't. I thought that was really interesting.
22. I kept being concerned about people's personal space and not stepping on them.
23. I was a little surprised at how connected I felt with the other people. I think, like the other gentleman was saying, somebody coughed, and I thought 'Am I too close to this person?' It was kind of strange.
24. In one way, I felt like there wasn't enough space. On the other hand, the constricted space, sort of, increases interactions and like, yeah, there was something happening. That was an interesting dichotomy for me.
25. What was interesting for me [was that] we couldn't see each other's body forms. At one stage I was lying down and at one stage I was kneeling, and I thought that was interesting. I was curious what everyone else was doing.
26. I was struck by how quite quickly something that was quite abstract—the light—suddenly held a lot of meaning when you perceived [them] as being people. I was quite struck when we all walked into the middle together, how that felt really real.
27. [?] get the proprioception, you know, to figure out how far you were from the other person and all that. But it was kind of fun, that was fun.
28. **Expected haptic feedback when connecting with creature and others, then felt it anyway, a little.**
29. **I enjoyed the subtle movements of the gloves. You felt aware of your body, but mostly more focused on the molecule.**
30. *It also felt really cool that I felt the heat from the light.*
31. *Whenever I would physically merge with any of the other participants, I got chills.*
32. *I loved touching people—I think because of the lockdown this enjoyment was even more heightened.*
33. *It also felt really cool to actually step inside others and also put [the molecule] in the ground.*
34. *Following the [dynamical molecular organism] with my nose, it felt like I was breathing it in.*
35. *When we were smoke.*
36. *I really enjoyed when we climbed on top of each other and all stood in the same spot.*
37. *I was struck with how quickly the abstract lights grew to hold tangible meaning, to understand them as other people. It was especially poignant when we moved toward the centre and felt as if we might collide or enter each other's personal space.*
38. *When bodies came together it felt like I was being tickled.*
39. ***Being able to get closer to people and become the same light. Knowing that we were all in different places having a shared experience that wasn't on Zoom.***

40. ***I was not sure if there was an effect when I touched or if someone else touched my heart light. In some ways I felt that I would have liked to be able to show my consent towards someone else interacting with my heart light.***

Theme: Ego Dissolution

1. For me, the whole feeling of just being in that space was that my identity felt lost. And then when you expand you feel like the [vastness] of the space that you're in and the energy that forms meant [for me] that myself as an identity didn't matter anymore and that was actually really liberating.
2. Yeah, it was very beautiful. I really loved how it challenges the relations. Like remember when we just gathered around, the worry at first [that we] would hurt one another, and then it quickly vanished. Now I just feel like it helps you dissociate from something within you. I think it can relate to what you said [participant name], about how your identity gets a bit lost. And I don't know if it for me was my identity or my anxieties that I put on the side. I feel present right now.
3. Identity didn't matter anymore; it was about experiencing things together. That was wonderful. There was an exterior manifestation of identity, yeah, it was new.
4. It was more like a space to be free, you could do whatever you want and not feel judged. A bit different from going to the gym.
5. I'm really enjoying [the current moment] when somebody talks, turning to them as though you're going to look in their face but you're just looking at this shape. And that feels really nice! Knowing that's a person and hearing your voice, and everything's live and real and connected, but then there is also this really abstract element. That's really fun.
6. I enjoyed not knowing who was who.
7. [The anonymity] Relaxing.
8. I feel like when you meet people outside of here there's so much superfluous stuff on top; there's layers of personality and layers of expectation that they also portray. Weirdly, here, when you're seeing someone just as a blob of light and two little blobs, which you can assume are hands, you lose all of that and you see more of a personality. I know that sounds weird. I almost see authentically. Like a childishness, like a pureness in it.
9. Takes down the ego a bit. I just mean once you're talking to a point of light, I think it produces that sense of the other person's inner sense and yours and, I guess, would allow you to talk in a more matter-of-fact way.
10. I found [the anonymity] quite powerful because we were all completely equal in the space. Any of the pre-judgements that come in from how people look, sound and that sort of thing just aren't there.
11. Yeah, I felt that also [when touching one another's heart centres] and, the sweetest tenderness or pure, childlike love in how stripped back, without any of the assumed layerings that we place upon reality and relationships and just, to the absolute core, it was truly beautiful. Thank you, all of you. Thank you, Isness.
12. It's a completely other way of connecting that I'm not familiar with. All the usual stuff disappears.
13. There was a freedom in being there.
14. Yeah, I think so. The fact that you can't see anybody and it's just you [trails off]. You can't really be self-conscious.
15. I felt a little self-conscious at the beginning when everyone's position was established but once everyone had moved around, I felt much less self-conscious.
16. **Everyone together moving arms from side to side was magic. We were all the same. We were all one. We all looked the same but had our own spirits.**
17. **Group dynamics – rejection, not being part of it, missing the point. Helpful to realize as we were all equal this was completely my filters. Able to let those anxieties go. Judgments of others based on voice and what they sound — slowly melting away.**
18. **In a time where the human race is being told to be socially distant, it is fantastic to have this representation of how connected we are in reality, and that having to stay physically apart doesn't prevent us from mentally and energetically connecting to each other. Also, that sometimes what actually prevents deeper connection is the outward assumptions we make of one another based on physical appearances and social conventions.**
19. **The experience was strange. It seemed to reduce relationships between people to the simplest concept—connectedness—but without visual cues and no judgements that go with it.**
20. **It's a lot of easier to focus on people when they are just 3 points. I felt a bit more able to understand and interact in the absence of extra cues. Maybe that's just my [asperges] talking though.**
21. **Love the smoke effect and the ego erasure.**

22. *The sense of connection with the other participants at a real level not superficial, societal, etc. Just them as they are.*
23. *That you could tell the personalities of people around you despite not being able to see them.*
24. *The sense that everyone was the same but with different energies and then the blending of these energies and the anonymisation of them as the session progressed.*
25. *I also liked the sandbox nature where we were anonymous and depicted only by points of light.*
26. *Feeling of anonymity, connection, playfulness, equality.*
27. *I enjoyed the sense of seeing others while not having to see the superficial form, which led to the felt sense of connection as beings.*
28. *The inability to perceive people as I normally would.*
29. *Loss of personal space. Being able to connect with other people as if touching was particularly enjoyable due to current COVID-19 restrictions. Meeting strangers as light forms with voices reduced preconceptions and helped us connect quickly, I think.*
30. ***Interacting with others' disembodied gestures had a quality of dissolving boundaries, gentleness, and warmth.***
31. ***It reminded me slightly of a trip, but mostly of a very intense and beautiful meditation session. The one big difference being the shared aspect of it. The connectedness of the group in the middle of a dissolved ego was really fascinating. Thank you!!!***

Theme: Supportive Setting

1. I loved [the chaperone line]...At the beginning I didn't really pay much attention to it, but by the end I was like 'the blue line makes me feel safe and really contained', both in physical space but, maybe because of that reliance, without really thinking about it, it made me feel like it emotionally or something as well. By the end, I was like 'I love the blue line!'. And then I was surprised by that.
2. The talk that you gave, [Facilitator's name], at the start was really helpful as well. Especially the David Bohm quote, I was really interested in the general gist of 'the luminosity of this is not really available to us with our current brain chemistry'.
3. **Really nice to have little bit of getting to know [?] (movements/dancing) at the start to warm up and make everyone comfortable. [Facilitator's name] is a brilliant host!**
4. **The smoke-light point was particularly nice. I very much enjoyed the mudra lights and the organism.**
5. **Nice space. The touching the ground thing was cool. I liked picking the molecules.**
6. **Camp fire start/end was a nice touch.**
7. **Lovely narration, visuals well produced with further R&D could be even better.**
8. **[Host Name]'s incense gave a real presence to the body-smoke. That worked REALLY WELL. Do that. A mid-way breeze cleared the room of incense for a bit and it really changed the experience. I was glad when it returned.**
9. **I loved the bit where the 'history trails' came to the creature. Made it feel a lot less spastic.**
10. **[My host] put incense on at the moment smoke appeared. I found that really enhance the experience.**
11. **I've been in several group meditation scenarios (in person) and always found them invasive and stifling. I often respond badly to 'energy-policing' that is sometime found in such environments. The Isness platform is comfortable and safe in a way typical group meditation environments often aren't.**
12. **Setting an intention – at first, I thought that this might be difficult to [reflect on connection with] strangers, but there was no pressure to give a long statement. I felt the breathing exercise helped you get comfortable in the space.**
13. **The repetition of the woman's voice helped situate you within the space. Gave you a focus.**
14. **I thought the guide did an excellent job of setting the tone without giving too much instructions or being intrusive.**
15. *I felt the guide was relaxed and not overpowering. [Facilitator's name] put me at ease.*
16. *I loved how gentle [Facilitator's name] was of a guide, the guided meditation script was on point, and the progression of activities and visuals were wonderful. I loved how in a matter of moments I felt a similar peak experience that I am more accustomed to experiencing with skilled dancers.*
17. *The guided meditation was super. Loved the woman's voice.*
18. *I really liked the opening and closing exercises with [Facilitator's name]. It really helped to hold the experience and make me feel more comfortable with everyone.*
19. *The reminders by the guide to be gentle with it because it held everyone's intentions was a lovely touch.*
20. *Loved the narration.*
21. *I enjoyed the quality of the guided meditation and the tone of voice of the facilitator.*
22. *The female narrator and overall experience was excellent, but I cannot say the best ever.*
23. *The sense of being present and absorbed by the experience. The music really helped with this. Great music!*
24. *The music and the gentle lighting.*
25. *The campfire start/end was a nice touch—it felt like other ceremonial experiences, and setting out on a journey.*
26. *The time of reflection at the end of the session, hearing other people's thoughts on the experience made the experience more meaningful.*
27. *Enjoyed the sharing of impressions with other participants in the end too.*
28. *Most of the visuals and audio were great. I really enjoyed the smoke effect, the spatial weirdness, and the interactions with the light creature.*

Theme: Sense of play

1. I had a similar experience, with realising the point isn't about trying to control it, the point is just to enjoy it and play. Yeah, I really enjoyed it.
2. There certainly was a sense of playfulness in this, which was really nice. As adults that's not always something that we pursue. It was good.
3. [Coalescing] felt nice, yeah. It felt lovely. There are points where [you wonder] 'are they playing with me?' and [I realised] 'yes, we're playing with each other'. It's a comfortable feeling.
4. I was just trying to make my own patterns and overwrite everything else, and come up with new ideas, and march through everything. That's me in a social situation.
5. With a lot of this, it was just so much fun playing with you guys. Which, I mean, I'm an adult, you sound like adults, that's not something that happens often.
6. I think there was a point where someone was drawing lines with traces and I couldn't help but draw some circles around their lines and start playing around like that. Almost like kids with sparklers, or something.
7. It was very interesting and engaging [to play with one another].
8. [Coalescing] was quite playful. That was quite fun.
9. I think it was playful to interact with people, almost not as people but as people in a different form, or maybe not as *physical* people, is what I mean. And to see these different energy balls moving around. That was different to what you could do otherwise, and fun.
10. I was inspired to dance on the top of the table at one point.
11. I enjoyed how playful it was. It reminded me how fun humans are in their capacity for play.
12. It reminded me a little of writing with fireworks, like when you had sparklers as a kid.
13. **I felt playful rather than deep.**
14. **Playful connection — a sense of love.**
15. **It was very playful, and that part was fun. It was very relaxing.**
16. **Felt very playful as we continued.**
17. **The changing mudra light was fun.**
18. *Playful exploration of myself in space and flowing into connections with others.*
19. *A feeling of being grounded, a playfulness facilitated in a calm way.*
20. *That there were a small number of participants, so we could interact in a small group. Everyone was friendly and playful.*
21. *The creative aspect. Drawing lines. Pressing my fingers to create energy.*
22. *It was very fun, very unique. I feel privileged to have had the opportunity to take part.*
23. *It was fun.*
24. ***I'm not a big gamer and hesitate to make a generalized comparison to multiplayer interactive games. While mutually interactive and peaceful, a similar experience might be reached through guided meditation together while holding hands with strangers with your eyes closed. The visuals were cool, and there was a degree of intimacy, and the technology was not 'in the way' but rather helping to connect in different locations, which is certainly cool. Overall, I thought it felt more like playing an interactive game with fun teammates than a spiritual experience.***

Theme: Transcendence of Space and Time

1. It was a funny feeling when the thing went through the floor. It felt like the space opened up to me. I'd been aware of the confines of [the space], vaguely. And then we pushed it through the floor and it was very very odd to feel like everything opened out.
2. [The molecule] was just so going down beneath the floor, I couldn't comprehend it.
3. I had no idea who anybody was...I have no idea where I am in the space so I'm quite interested, when I open my eyes, where on Earth I'll be.
4. [The molecule] going into the ground, that's just so fascinating that you can overcome the physical boundaries of our reality, that always blows my mind.
5. I think I was imagining things didn't have the same human scale, so at times it felt like this could be a ginormous space and we were a completely different scale, or it could be a microscopic space, and it didn't really matter, actually.
6. I've lost perception of time.
7. **It was the beginning. I didn't feel like there was an end. Actually thought I was in space.**
8. **I couldn't believe how much my perspective changed when we put the molecule into the ground.**
9. **It seemed divorced from space and time. I had a hard time giving in to the concept without trying to analyze what was going on. Very enjoyable experience.**
10. **I didn't feel a sense of time. I was surprised at the end that it had been an hour.**
11. *I liked being unaware of restrictions like time.*
12. *Looking down and seeing the world under the floor.*
13. *I liked moving the object and unravelling and when we put it through the floor. I liked the parts that allowed me to view the world in a way that isn't possible in real life.*

Theme: Noetic Quality

1. I got quite emotional, for a moment. And then the experience was guiding us forward, so it was fleeting. But I got this surge of emotion where I don't know if I wanted to gasp or cry or what it was, but I was kind of shocked in awe.
2. That's definitely a spiritual experience of some sort. It's, like, tangible.
3. I didn't feel any nervousness. Seeing all the lights together reminded me of a dream I had years ago where the universe was ending and everybody was dissolving into balls of light and merging into one. So, I was like 'oh cool! The universe is ending. I'm cool with that'.
4. **Like awakening from a most blissful dream, that leaves its loving traces in body, heart and mind. A remembrance, a return to the simplicity and purity of being, that which we always are and can at time forget. Thank you for the remembrance.**
5. **The power and light in each individual — how this can be more potent with guidance and moving together.**
6. **Having everyone be portrayed as balls of light seemed like a very true representation of a person. Just sort of struck home as a true way to see someone.**
7. **I enjoyed intermingling lights with everyone. Got a little teary-eyed during that part, I miss people so much with COVID. And making those connections felt quite profound.**
8. *Pure unity.*
9. *I really did gather a DMT/death like Becoming for my reality. The experience felt fun, fleeting, VERY special, and at times holy. I would not say that in all my teachings and explorations that this ceremony dissolved ego in the sense that one can achieve in Healing and with inward work. This experience felt galactic, MUCH bigger in terms of the sense of Soulfulness, meaning after death, then what?*
10. *The abstraction and the time given to becoming connected to that abstraction.*
11. *The distillation of existence.*

Theme: Comparison to Other Altered States

1. [Taking psychedelics] wasn’t quite like this, but I guess there’s elements of it, for sure.
2. **Similar to experiences that I have had as somatic visions through medicine plants. The interconnective nature of energy/intention and the ‘strings’ that appear to interconnect us all with all living matter. This also related to childhood dreams I had prior to any ‘psychedelic experience’.**
3. **About 5 days ago I tried mushrooms for the first time – if I hadn’t have done that I could have selected 5 for most of them (most I’ve ever felt lost sense of time/space/ego).**
4. **It was like psychedelics without the psychedelics.**
5. **I wonder [how] people with and without psychedelic experiences relate to Isness.**
6. **There was something common with psychedelic experience.**
7. *I experienced the sense of interconnectedness that I would I've only previously noticed with the help of psychedelics in the right setting.*
8. *The experience brought up memories for me of medicine plant journeys and realisations that have shaped my life and what give my life meaning. These realisations are very much connected with going beyond form and finery conceptions, and also beyond mainstream conceptions of leading a good life.*
9. *Felt psychedelic.*
10. **I wondered before and during if one's prior experience with meditation and psychedelics significantly impacts how one experiences Isness. I've got some experience with both and didn't feel ‘blown away’ by the ceremony, although I did enjoy it and feel it has potential. I did not feel I was being critical or judgmental during it, I was simply experiencing it. I did feel more peaceful / less agitated and the end and connected to the others. Everyone was kind, sweet, and playful.**

Theme: Sense of Beauty

1. When I let go of needing to have control over [the molecule], it actually became spectacularly beautiful. And, actually, sitting motionless and watching what else was going on. And there was a bit, I think, where [participant's names] were dancing and interacting and it was so beautiful. So yeah, there was something about letting go of control.
2. I was amazed [by] how moved I was. I think it was the juxtaposition of the frustration and then the beholding beauty. I think the music really helped. It felt very beautiful to sit back and witness. I don't normally get moved quite so much.
3. [Molecules] are so beautiful.
4. It felt quite powerful and quite beautiful, really. The idea of interacting with abstract entities but were at the same time real entities, just not physically in this space, yet at the same time visible, and touchable, and present.
5. Yes, thank you. I was a little bit doubtful in the beginning, I didn't know what to expect and what was going to happen. It was a beautiful experience.
6. ...the whole sort of connectedness of it. The sort of weird amorphous space that you end up taking up and then just the mingling with everybody and the absence of borders and things like that...was just a very very beautiful experience.
7. **Respect, awe, love for the energy being — felt its innocence! And beauty.**
8. **The connector object is beautiful when it opens but seems too flickering when in the middle. Reminds me of flash [illegible] when I have a migraine.**
9. ***Very beautiful visuals, audio and emotions that came with it!***

Theme: Reflection on Mortality

1. It sounds really cheesy, but it's given me a deep understanding of maybe what happens after life ends. I feel a bit more connected to those people that have gone, having now seen this.
2. It's clear that the energy isn't just gone, it goes somewhere. It's beautiful to think that it's all out there somewhere and it's still circling you constantly.
3. [I feel] sad that [the lights are] going away. [I feel] a lot more relaxed than I felt when I came in.
4. [When the lights faded] Does this mean we're dead?
5. There was one moment, we were talking about seeing past energies and I was thinking about my cat, whom I lost last year. I really miss her. I could really see her and the [?] was writing her name. It was beautiful, thank you.
6. **That was super cool! I arrived feeling pretty anxious and disconnected from others, but after that experience I feel much more calm and hopeful. Hugely due to the focus of how energy doesn't truly disappear. I guess the fear of dying (the existential threat) is always on our minds but that experience quietened the fear. AMAZING!**
7. **On the occasions where thing went dark, I felt a little triggered. Like, OMG, everyone's gone! Or, like my senses disappeared and so I couldn't perceive anyone. Which was lonely! Noticed some agitation due to [Participant Name] having glitches, but then I was equating it to the idea of Boudicca. Because being light being felt akin to the idea of enlightenment, I thought how if anyone gets left behind it's going to leave lingering unhappiness for everyone. At one point, the energy web circle broke on [Participant Name]'s side. I wanted to mend it but couldn't seem to. Made me a little sad – for [Participant Name] and for all of us.**
8. **When meditating upon the traces of energy remaining around us (fave bit!) I felt, again, the realization of time being not linear; I felt like I was summoning a Patronus from my lost loved ones. I think this would be amazing for grief counselling.**

Isness pre-ceremony questionnaire

Start of Block: Introduction

Q23 On the following pages are a few quick questions for you to complete before starting the Isness virtual reality ceremony. Please read the questions carefully.

Thank you!

End of Block: Introduction

Start of Block: Unique code

Q26 Please enter a unique code / text (e.g. yellowsubmarine123). You will need to enter the same information on a second form after Isness has finished - this is so we can link your data and keep you anonymous.

____

End of Block: Unique code

Start of Block: Demographics

Q28 What country are you participating from?

____

Q27 What time is your Isness session due to start? Please write as time and your time zone (e.g. 5pm GMT)

____

Q29 What is today's date? (format required is DD/MM/YYYY)

____

End of Block: Demographics

Start of Block: Inclusion of Other in the Self (IOS) scale.

Q14 Below are a series of circles. The circle on the left of the pair represents you, while the circle on the right represents the other people who will be participating in your virtual reality Isness experience (who you've not yet met).

Which picture best describes your relationship with the other Isness participants?

○ a (4)

○ b (5)

○ c (6)

○ d (7)

○ e (8)

○ f (9)

End of Block: Inclusion of Other in the Self (IOS).

Start of Block: Openness to the upcoming experience scale

Q17 Please rate to what extent the statements below apply to your feeling right now, **at this present moment.**

On the scale below each statement, mark with an "X" a number between 0 and 100, where 0 = Strongly disagree and 100 = Strongly agree. There are no right or wrong answers. Do not spend too much time on any one statement but give the answer which seems to best describe your present feelings. This is a survey which is used for personal development ceremonies, so some of the questions may not be too relevant for the virtual reality experience you are about to have.

Strongly disagree Strongly agree

0 10 20 30 40 50 60 70 80 90 100

I am preoccupied with my work and/or life duties. ()	
I feel open to the upcoming experience. ()	
I feel well prepared for the upcoming experience. ()	
I have a clear intention for the upcoming experience. ()	
I feel comfortable about the upcoming experience. ()	
I have strong expectations for the upcoming experience. ()	
I am in a good mood. ()	
I feel anxious. ()	
The environment/setting feels good for my upcoming experience. ()	
I have a good feeling about my relationship with the group/people who will be with me during my experience. ()	
I have a good relationship with the main person/people who will look after me during the upcoming experience. ()	
I feel able to receive without fighting back. ()	
I have a lot of things I should be doing right now. ()	
I have a lot on my mind right now. ()	
I feel I have endless time to devote to this experience. ()	

Page Break

Q9 Thank you for your responses - Isness will start soon.

End of Block Openness to the upcoming experience scale

Isness post-ceremony questionnaire

Start of Block: Introduction

Q26 Thank you for taking part in Isness. There are now some further questions for you to answer about your experience.

Page Break

Q27 Please enter the unique code / text you created in the pre-Isness survey (e.g. yellowsubmarine123).

____

End of Block: Introduction

Start of Block: (ii) Peak experiences questionnaire

Q18

The following questions are about your memory of the Isness ceremony experience. Try to think of the most intense part, the peak of the experience.

Not at all						Very much so				
0	10	20	30	40	50	60	70	80	90	100

1. I remember my emotional experience clearly, and with confidence could describe how I felt. ()	
2. I remember my surroundings exactly and with confidence can recall what the environment looked like. ()	
3. The peak experience exists in my mind like a very clear photograph or video. ()	

End of Block: (ii) Peak experiences questionnaire

Start of Block: (iv) Communitas scale

Q19

Please indicate how strongly you agree or disagree with the following statements about your Isness ceremony experience, taking into account that 1=Strongly disagree and 7= Strongly agree

Strongly disagree			Neither agree nor disagree			Strongly agree
1	2	3	4	5	6	7

During the ceremony, I felt a bond with my fellow participants that I could not experience outside the ceremony. ()	
During the ceremony, I felt a sense of belonging with the other participants. ()	
During the ceremony, I felt a sense of harmony with the others. ()	
During the ceremony, I felt a sense of sharing with the other participants. ()	
The ceremony really allowed me to get to know the other participants. ()	
During the ceremony, I felt that social status became irrelevant. ()	
During the ceremony, I experienced all participants as equal based on basic shared humanity. ()	
During the ceremony, ego-related tensions between participants seemed to resolve. ()	
During the ceremony, I felt a strong connection to another participant. ()	
During the ceremony, I felt a strong connection the facilitator. ()	

End of Block: (iv) Communitas scale

Start of Block: (v) MEQ 30

Q27

For each of the following, please rate the extent to which you agree with each statement. Looking back on the entirety of your ceremony experience, please rate the degree to which

at any time you experienced the following phenomena. Answer each question according to your feelings, thoughts, and experiences at the time of the ceremony.

	None, not at all	So slight cannot decide	Slight	Moderate	Strong (equivalent in degree to any other strong experience)	Extreme (more than any other time in my life and stronger than 4)
	0	1	2	3	4	5

1. Loss of your usual sense of time. ()	
2. Experience of amazement. ()	
3. Sense that the experience cannot be described adequately in words. ()	
4. Gain of insightful knowledge experienced at an intuitive level. ()	
5. Feeling that you experienced eternity or infinity. ()	
6. Experience of oneness or unity with objects and/or persons perceived in your surroundings. ()	
7. Loss of your usual sense of space. ()	
8. Feelings of tenderness and gentleness. ()	
9. Certainty of encounter with ultimate reality (in the sense of being able to “know” and “see” what is really real at some point during your experience. ()	
10. Feeling that you could not do justice to your experience by describing it in words. ()	
11. Loss of usual awareness of where you were. ()	
12. Feelings of peace and tranquility. ()	
13. Sense of being “outside of” time, beyond past and future. ()	
14. Freedom from the limitations of your personal self and feeling a unity or bond with what was felt to be greater than your personal self. ()	
15. Sense of being at a spiritual height. ()	
16. Experience of pure being and pure awareness (beyond the world of sense impressions). ()	

17. Experience of ecstasy. ()	
18. Experience of the insight that “all is One”. ()	
19. Being in a realm with no space boundaries. ()	
20. Experience of oneness in relation to an “inner world” within. ()	
21. Sense of reverence. ()	
22. Experience of timelessness. ()	
23. You are convinced now, as you look back on your experience, that in it you encountered ultimate reality (i.e., that you “knew” and “saw” what was really real). ()	
24. Feeling that you experienced something profoundly sacred and holy. ()	
25. Awareness of the life or living presence in all things. ()	
26. Experience of the fusion of your personal self into a larger whole ()	
27. Sense of awe or awesomeness. ()	
28. Experience of unity with ultimate reality. ()	
29. Feeling that it would be difficult to communicate your own experience to others who have not had similar experiences. ()	
30. Feelings of joy. ()	

End of Block: (v) MEQ 30

Start of Block: (vi) Ego-dissolution inventory (EDI) (Nour et al., 2016)

Q20

Please rate to what extent the following statements apply to your Isness ceremony experience by choosing a number between 0 and 100 on the scale below each statement. Note that zero corresponds to your normal waking consciousness.

	No, not more than usually						Yes, I experienced this completely			
0	10	20	30	40	50	60	70	80	90	100

I felt especially assertive. ()	
I experienced a dissolution of my "self" or ego. ()	
I felt more important or special than others. ()	
I felt at one with the universe. ()	
My ego felt inflated. ()	
I felt a sense of union with others. ()	
I felt especially sure-of-myself. ()	
I experienced a decrease in my sense of self-importance. ()	
I felt especially keen and competitive. ()	
I experienced a disintegration of my "self" or ego. ()	
I felt my viewpoint was worth more than other peoples' ()	
I felt far less absorbed by my own issues and concerns. ()	
I felt especially self-confident. ()	
I lost all sense of ego. ()	
I felt especially self-assured. ()	
All notion of self and identity dissolved away. ()	

End of Block: (vi) Ego-Dissolution Inventory (EDI) (Nour et al., 2016)

Start of Block: (x) Inclusion of Other in the Self (IOS)

Q37 Below are a series of circles. The circle on the left of the pair represents you, while the circle on the right represents the other people participating in your virtual reality Isness experience

Which picture best describes your relationship with the other Isness participants?

- ○ a (4)
- ○ b (5)
- ○ c (6)
- ○ d (7)
- ○ e (8)
- ○ f (9)

End of Block: (x) Inclusion of Other in the Self (IOS)

Start of Block: (xii)

Q21

Please answer according to your feelings towards your Isness ceremony group, taking into account that 1=Not at all and 7= All of the time

	Not at all	Almost never	Occasionally	Sometimes	Frequently	Almost always	All of the time
	1	2	3	4	5	6	7

I felt that we were one ()	
I felt more sensitive to my emotions and feelings because I was surrounded by people who felt the same. ()	
I felt a strong shared emotion. ()	
I felt really united with the group, almost melded into one. ()	
I felt more intense emotions because we all went through the same experience. ()	

End of Block: (xii)

Start of Block: (xiii) Bodily effects

Q22

Have you experienced any of these bodily effects during the ceremony? Tick all that apply.

- ▢ Nausea or vomiting (1)
- ▢ Diarrhea (2)
- ▢ Body aches (3)
- ▢ Ringing in the ears (4)
- ▢ Headache (5)
- ▢ Sweats or chills (6)
- ▢ Trembling (7)
- ▢ Crying (8)

End of Block: (xiii) Bodily effects

Start of Block: Demographics

Q11 Who is your host for the experience?

- Vadim (4)
- Dave (5)
- Rhos (6)
- Olivia (7)
- Mike (8)
- Greg (9)
- Justin (10)
- Harrison (11)
- Jeremy (12)
- Matthew (13)
- Jamie (14)
- Rachel (15)
- Sara (16)
- Joe (17)

Page Break

Q13 What is your age in years?

____

Q13 What is your gender?

- ○ Male (1)
- ○ Female (2)
- ○ Other (4)
- ○ Decline to state (5)

Page Break

*

Q12 How many times have you had a virtual reality experience in the past 12 months?

____

Q25 Have you taken part in Isness before?

- ○ Yes (1)
- ○ No (2)

End of Block: Demographics

Start of Block: Final comments

Q29 Were there any technical problems that you noticed? If so, how did these impact the experience?

Q30 What did you *like* about the experience?

Q31 What did you *dislike* about the experience?

Q32 How do you think the experience could be *improved*?

____

____

____

____

____

Q33 Yes - the whole time (1)

- ○ Yes - some of the time (4)
- ○ No (2)
- ○ Unsure (3)

Q34 How, if at all, did their presence / absence impact your experience?

____

____

____

____

____

Q28 Do you have any other comments about the experience?

If you would prefer, your host will have paper and pen (please write your unique code at the top of the paper so we can link your data).

____

____

____

____

____

End of Block: Final comments

Start of Block: Debrief

Q10 **Debrief**

Thank you for taking part in Isness.

Isness is a multi-person virtual reality experience that we have created. Here we are investigating how running Isness in a distributed (across multiple sites) manner impacts the Isness experience. We are particularly interested in the impact of Isness on feelings of connectedness between participants.

If you have any further questions, please do not hesitate to get in touch with the research team
Dave Glowacki (glowacki@bristol.ac.uk) or Olivia Maynard (Olivia.maynard@bristol.ac.uk).

If you would like the study team to contact you with details of this study or other work they're doing, you can enter your email address at the following link - this separates your email address from your study data, keeping your responses anonymous

https://bristolexppsych.eu.qualtrics.com/jfe/form/SV_6mSi4TBYy0Ir9CR

End of Block: Debrief
